# COVID-19 dynamics in Madrid (Spain): A new convolutional model to find out the missing information during the first three waves

**DOI:** 10.1371/journal.pone.0279080

**Published:** 2022-12-22

**Authors:** Efrén M. Benavides, María Ordobás Gavín, Raúl Mallaina García, Sara de Miguel García, Maira Ortíz Pinto, Ramón Doménech Gimenez, Ana Gandarillas Grande

**Affiliations:** 1 Department of Fluid Mechanics and Aersospace Propulsion, Universidad Politécnica de Madrid, Madrid, Spain; 2 Epidemiology Department, Directorate General of Public Health, Madrid Regional Health Authority, Madrid, Spain; 3 Strategic Planning Department, Directorate of Integrated Healthcare Process, Foundation on Innovation and Research in Primary Care Foundation FIIBAP, Madrid, Spain; Xavier University, UNITED STATES

## Abstract

This article presents a novel mathematical model to describe the spread of an infectious disease in the presence of social and health events: it uses 15 compartments, 7 convolution integrals and 4 types of infected individuals, asymptomatic, mild, moderate and severe. A unique feature of this work is that the convolutions and the compartments have been selected to maximize the number of independent input parameters, leading to a 56-parameter model where only one had to evolve over time. The results show that 1) the proposed mathematical model is flexible and robust enough to describe the complex dynamic of the pandemic during the first three waves of the COVID-19 spread in the region of Madrid (Spain) and 2) the proposed model allows us to calculate the number of asymptomatic individuals and the number of persons who presented antibodies during the first waves. The study shows that the following results are compatible with the reported data: close to 28% of the infected individuals were asymptomatic during the three waves, close to 29% of asymptomatic individuals were detected during the subsequent waves and close to 26% of the Madrid population had antibodies at the end of the third wave. This calculated number of persons with antibodies is in great agreement with four direct measurements obtained from an independent sero-epidemiological research. In addition, six calculated curves (total number of confirmed cases, asymptomatic who are confirmed as positive, hospital admissions and discharges and intensive care units admissions) show good agreement with data from an epidemiological surveillance database.

## 1. Introduction

Since the COVID-19 disease appeared in the city of Wuhan (China) in December 2019, firstly as a cluster of pneumonia cases [1] and secondly as a novel strain of coronavirus [2], it has struck every corner in the world in a process where the virus has shown its ability to spread quickly by means of a person-to-person transmission. In this process, mainly during the onset of the pandemic, the unrestricted movements of asymptomatic and mildly infected people were underestimated and the propagation rate exploded in many countries at the same time. The authorities’ response was a generalized ‘lockdown’. These interventions from authorities started on 23^rd^ January with a Chinese government’s order for Wuhan to enter lockdown, which was in the next 24 hours extended to all the Hubei province.

Meantime, the virus SARS-CoV-2 and its associated disease COVID-19, spread to Europe, where it intensified quickly, firstly in Italy [3] and secondly in Spain [4]. The first imported Spanish case of COVID-19 was detected on 31^st^ January 2020 and the first case of local transmission was identified on 26^th^ February 2020 [4]. After that, from the end of February, the number of cases increased exponentially, peaking on 20^th^ March. This peak occurred 5 days after the declaration of State of Alarm and the implementation of the national lockdown. During the following 6 weeks, there was a sustained decline in the number of COVID-19 cases. This first wave was followed by three others with maximum incidence peaks in early November 2020, mid-January 2021 and mid-April 2021 [5]. Madrid, as an important Spanish hub for public transportation with nearly 6.8 million people and a huge amount of foreign travellers, also suffered a severe first wave. Later, the epidemiological trend was similar to the national one, showing 4 epidemic waves. The characteristics of all of them show differences in terms of age groups, severity and evolution and were affected by national and regional restrictions [6].

The Spanish Government focused its strategy on slowing down people movements (by imposing severe restrictions on non-essential public and private transportations) and on improving the identification and isolation of infected individuals (thanks to trace and test suspected cases). However, additional social distancing measures had to be applied in order to slow the growth of the outbreak: among them, the permanent use of face masks and the reduction of contacts between non-cohabiting individuals. These harsh measures were the response to an unexpected factor: the epidemic spread too fast. The suspicion that there was a community transmission due to a strong presence of asymptomatic individuals became important as it seemed to be the simple explanation of that fact. That is, there was a strong evidence to suggest that there were a significant fraction of asymptomatic individuals [7, 8] who could still transmit infection [9]. Hence, the Government priority moved to generalize the test to all the population in order to identify and isolate asymptomatic carriers. Altogether, COVID-19 has shown the importance of mathematically describing the spread of the disease in the presence of complex social behaviour and different responses of infected individuals.

Taking into consideration the previous facts, it seemed reasonable to build a mathematical model with several types of infected individuals. Every group should have its own transmission rate, and, more important, its own list of health events (symptom development, hospital admission, ICU admission, recovery, death…). For example, an asymptomatic individual will never end in a hospital whereas a severely infected individual will undergo the event of being admitted to an intensive care unit (ICU). In addition, two severe infected individuals who were infected the same day will spend a different amount of time in recovery, which, indeed, brings up the necessity of introducing probability distributions into the description of such events. This also requires writing down the number of individuals who share the same infection date and distinguishing when they are going to suffer a future event. The mathematical tool in charge of this work is the convolution, which introduces integrals into the differential equations and makes a clear distinction from any standard susceptible-infective-recovered (SIR) model (which uses ordinary differential equations without any convolution integral). The introduction of convolution integrals opens the door to a completely new set of mathematical descriptions [10–17]. In particular, Reference [17] illustrates how a convolution integral can be used to enhance a SIR model with a general infectious period distribution. However, the approach in this article will be different because it will add an integral operator without removing the classical derivative operator. This will add flexibility and robustness to the model. In particular, it will allow us to use the infection date as a trigger signal and, along with convolution integrals, distribute the individuals over time to keep track of health events (such as development of symptoms, hospital admission, ICU admission and recovery or death).

A convolutional model with only three types of infection, mild, moderate and severe, was used to make predictions in the early stage of the Spanish outbreak, more precisely, in the first wave [15, 16]. This preliminary study helped to discover some promising characteristics of the model. In particular, it pointed out that the convolution integral allows isolation of the infection rate from the recovery rate, which has several advantages. Firstly, it permits making a compartmental model pertaining to the health events which, depending on the severity of the symptoms, have different effects. Secondly, it allows us to fix the median and the interquartile range (IQR) for those events only pertaining to aspects of the disease itself. Lastly, it allows extrapolation of mobility parameters from past scenarios to future scenarios without having to change the medians and the IQRs. This paper aims to improve such a mathematical model by including asymptomatic individuals in order to enhance the prediction capabilities.

A second objective of the paper is to use the independence between social and health parameters (ensured by how the model has been conceived) to obtain unknown information, such as, 1) the number of asymptomatic individuals who were present during the first wave in Madrid or 2) how governmental restrictions affected the evolution of the pandemic in this region. Independence means that changing one parameter over time does not necessarily require changing the others; the other way around, dependence means that changing one parameter over time does necessarily require changing at the same time some of the others. In this article, this ensures that if one parameter is independent of time (such as the time required for hospital admission, which is assumed to be the same for the first wave as for the last), then the changing of other parameter over time (such as the social mobility, which is clearly different for the first wave than for the later one) does not force the first to change. In the presented model, this independence is achieved due to a novel selection of the convolution integrals. This novelty makes a clear distinction with respect to other previous models presented in the literature [10–13].

To achieve the aforementioned objectives, the paper is structured as follows. Firstly, a background about available mathematical models is presented. Then, the new model is introduced as a way of enhancing flexibility and robustness. Secondly, epidemiological surveillance data from Public Health in Madrid on confirmed, asymptomatic, hospitalized, ICU admitted and deceased individuals are presented and used to fit some of the model parameters. Since officially documented data were more complete during the second and third waves and less during the first wave (because the asymptomatic were not being detected during the first wave: by 31^st^ March less than 1% of the Madrid’s population had been tested and by 6^th^ August less than 10% of the Madrid’s population had been tested [4–6]), the uncertainty is larger at the beginning. For this reason, the last two waves have been used to adjust the parameters and, as a result of the calculation, the evolution of the asymptomatic individuals during the early stage of the outbreak is obtained. Note that, this way, the mathematical model has been used to reconstruct missing information: mainly, the number of individuals who were asymptomatic and who presented antibodies.

## 2. Method and model

### 2.1 Background

The mathematical description of these kinds of propagation processes is based on several nonlinear differential equations which prescribe the evolution of the main agents. In its simplest form, the model only requires two frequencies, the transmission factor *b* and the recovery factor *a*, and three agents, Susceptible (*S*(*t*)), Infective (*Inf*(*t*)) and Recovered (*R*(*t*)), where the Infective are those individuals who have been infected and are able to retransmit the infection. Thus, the basic Susceptible-Infective-Recovered model (SIR) could look like this [17]:

$$\begin{array}{ll} \frac{dS}{dt} & =-b\frac{S}{N}Inf\\ \frac{dInf}{dt} & =\left(b\frac{S}{N}-a\right)Inf\\ \frac{dR}{dt} & =a\;Inf\\ N & =S+Inf+R \end{array}$$ (1)

This model has been used to study the Hong-Kong Flu [18] and can be applied to any transmittable disease, in particular, to COVID-19 [19–22]. Reader can find a complete description of these models in references [17, 23–26]. The meaning of the frequencies *b* and *a* as “production” and “destruction” rates are similar to those used in such references and comes from interpreting the terms on the right hand side of system (1): *b* is the probability per unit of time that an infectious person has of transmitting the disease to a susceptible one, and *a* is the probability per unit of time that an infectious person has of recovering. If the population *N* is constant and known, the last equation in (1) can be used to calculate *R*(*t*) and hence the third equation can be removed from the calculation. In addition, Ref. [17] introduces two new functions in system (1): the fraction of individuals who are still infective a time *τ* after having become infected as the function *P*(*τ*) and the number of individuals who were infective initially at *t* = 0 and are still infective at time *t* as the function *Inf*_0_(*t*), This way, the model (1) can be replaced by the following two equations:

$$\begin{array}{ll} \frac{dS}{dt} & =-b\left(t\right)\frac{S\left(t\right)}{N}Inf\left(t\right)\\ Inf\left(t\right) & =Inf_{0}\left(t\right)+\overset{t}{\underset{0}{\int }}\left[-\frac{dS}{dt}\left(t-\tau \right)\right]P\left(\tau \right)d\tau \end{array}$$ (2)

The last term in (2) is a convolution integral, which has been used to substitute the second differential equation in (1) with an integral equation. As long as function *P*(*t*) can introduce more than one model parameters, the model in (2) is more flexible than the model in (1); however, any social evolution with enough complexity will require *b*(*t*) to be a function of time and hence the parameters inside *P*(*t*) will be severely dependent on *b*(*t*). This severe dependency will drastically reduce the robustness and the prediction capability of the mathematical model.

In order to increase the flexibility and hence the ability to fit more complex and real scenarios of propagation, the simple models in (1) and (2) should be modified. As the pandemic has shown, this feature is crucial as long as many health and social events have, indeed, perturbed the evolution of the spread. As is well-known [17, 23–26], one way to add flexibility to the model written in system (1) is to increase the number of involved agents (compartments). For example, reference [27] considers a Susceptible group, which comprises healthy people, an Asymptomatic group, which collects infected individuals in the early stage of infection (who may infect susceptible individuals through droplets or direct contact), a Symptomatic unreported group, which comprises infected individuals not detected by the health system as a Covid case, a Symptomatic reported group, which comprises infected individuals detected by the system, and a Recovered group, which comprises individuals with temporal immunity. The resulting model [27] adds mathematical flexibility to the classical SIR model thanks to the new groups and the new parameters. However, as it has been previously discussed for model (2), having more parameters does not necessarily imply having a more robust description of a general pandemic situation.

Other models with less [28–32] or more [33–40] compartments can be built since the only required input to build the model is a flow chart with the agents and their corresponding production and destruction rates. For example, it is possible to incorporate a group of exposed persons (E) between the S group and the I group to obtain a SEIR model [17, 23, 24, 29, 32, 33, 35–37, 39–41]. In the same way it is also possible to include a quarantine group to obtain a SIQR model [17, 42]. Therefore, there is no limitation to the number of groups, which makes these kinds of models versatile enough to deal with the real propagation: populations can be grouped by age [33], weight, mobility, severity of symptoms, degree of immunity, etc. For a mathematical model including asymptomatic individuals structured by age of infection, see e.g. [43]. The larger the number of involved frequencies, the larger the number of complex scenarios which can be solved; however, in contrast, it also increases the number of dependencies between the frequencies (normally, if one frequency is changed in order to model a particular evolution, all of them must be changed), what forces to use machine-learning algorithms [44] and makes difficult the extrapolation of such frequencies to other future scenarios.

Flexibility can also be obtained by means of fractional derivatives. In these models [41, 45, 46], the first order derivatives are substituted by Caputo fractional derivatives of order less than one, where the order can be modified to obtain a better fit between the calculated curves and the actual ones. Since these models only change the mathematical operator associated to the derivative, all the previous models are susceptible to being transformed into its fractional version. From the practical point of view, the number of required input values is increased by the number of fractional derivatives. For example, if the integer derivative model with two input frequencies given by system (1) had to be transformed into its fractional derivative version, two new input parameters would have to be available to change the shape of the calculated evolution, thus, leading to a four-parameter model. However, it is difficult to link the fractional derivative order with a “measured” or known value, such as, for example, the average number of days that a severely infected person will need to recover from the illness. The reasoning is that there is not a straightforward connection between the mathematical number “derivative order” and any of the frequencies that describes the propagation. Thus, the meanings of the new parameters are not easily described using terms related to social and health characteristics of the studied population.

This article also aims to increase the model’s flexibility by the addition of new parameters, but the approach will be different from the previous ones. The main idea used in this article consists of substituting some of the frequencies by distribution functions, so that the production and destruction terms can be replaced by convolution integrals, but with the novelty of explicitly imposing the maximum independence between all the parameters. The outcome should be a model with more independent parameters, and hence, more flexibility and robustness. A first exploration of the possibilities of this idea to predict the spread of COVID-19 in Spain was presented in references [15, 16], which showed a promising perspective. Other researches [10–13] use a similar approach based on convolution or renewal integrals [14].

### 2.2 Basic convolutional model

To highlight the main novelty that the presented model exhibits, we will transform the basic standard SIR model written in (1) into its convolutional version. The main required groups are:

- *S*(*t*): Susceptibles. Those individuals who are healthy and can be infected at time *t*.
- *I*(*t*): Infected. Those individuals who have been infected at any time previous to time *t*.
- *R*(*t*): Removed. In this model, those individuals who are immune after having been infected and recovered or dead. They do not propagate the virus anymore.
- *Inf*(*t*) = *I*(*t*) − *R*(*t*): Infectious. Those infective individuals who have been infected and are able to transmit the infection. They were infected at some particular date in the past and are not recovered or dead yet at time *t*.

The convolution integral relates to the recovered or dead individuals at time *t* with the infected ones at any past time *τ* ≤ *t* and this consideration transforms the SIR model given in (1) into its convolutional version (C-SIR model), which is:

$$\begin{array}{ll} \frac{dS}{dt} & =-b\frac{S}{N}\left(I-R\right)\\ \frac{dI}{dt} & =b\frac{S}{N}\left(I-R\right)\\ \frac{dR}{dt} & =\int_{0}^{t}f\left(t-\tau \right)\frac{dI\left(\tau \right)}{d\tau }d\tau \\ N & =S+I \end{array}$$ (3)

Here, function *f*(*t*) is a distribution function modelling the fraction of infected persons who were infected at time 0 and became recovered in the period of time between *t* and *t* + *dt* as *f*(*t*)*dt*. Therefore, if *dI*(*τ*) is the number of individuals infected during the period (*τ*, *τ* + *dτ*), then *f*(*t* − *τ*)*dI*(*τ*)*dt* is the number of such individuals recovered during the period (*t*, *t* + *dt*). Finally, the convolution integral $dR=dt\int_{0}^{t}f\left(t-\tau \right)dI\left(\tau \right)=dt\int_{0}^{t}f\left(t-\tau \right)\frac{dI\left(\tau \right)}{d\tau }d\tau$ accounts for the total number of individuals infected during the period (0, *t*) and recovered during the period (*t*, *t* + *dt*).

A main difference with respect to models (1) and (2) is that the equation required to calculate *R*(*t*) cannot be dropped anymore. The reason to write the second equation using the derivative of the infected individuals $\frac{dI}{dt}$ instead of the derivative of the infectives $\frac{d\;Inf}{dt}$, as is usually done [17], is that, this way, the independence between the frequency *b* and the parameters which define the probability distribution *f* is explicitly imposed in the convolution integral. From a practical point of view, this is a significant improvement because the mathematical model is now more robust: it has enhanced its prediction capabilities. The key point in (3) is that it has been written in a way that allows an increase in the number of model parameters and, at the same time, preserve their independence. Now, there are at least three independent parameters in (3) which remains independent for any time *t*: *b*, which is still the “production” rate, and the parameters which determine the shape of the probability distribution *f*(*t*), which are the median and the interquartile range (IQR) in the case of using a Weibull distribution for the recovery time (see S1 Text). This is a crucial point of this model since, as it is later calculated, the social constraints will make the production rate (related to the stringency index in Fig 9) to have a strong dependence on time while the parameters related to the disease are kept constant over time.

The process employed to transform the standard SIR model (1) to the C-SIR model (3) can be replicated for any desired number of compartments or equations. There is not any practical restriction to transform a SEIR or a more complex model into its convolutional version (C- Version). In this article, we will use 15 compartments and 7 convolutions. For each new convolution we have to give a new distribution function *f*. For example, in order to account for the number of ICU admissions, we have to introduce the convolution integral $dt\int_{0}^{t}f_{ICUadm}\left(t-\tau \right)\frac{dI\left(\tau \right)}{d\tau }d\tau$, where *f*_*ICUadm*_ (*t* − *τ*)*dI*(*τ*)*dt* is the number of individuals who were infected during the period (*τ*, *τ* + *dτ*) and admitted in the ICU during the period (*t*, *t* + *dt*). Therefore, the full model replicates the equations given in system (3) (which already implements all the relevant novelties) many times. The following section and the S1 Text document contains a more detailed explanation.

### 2.3 Convolutional model for the COVID-19

**Fig 1:**
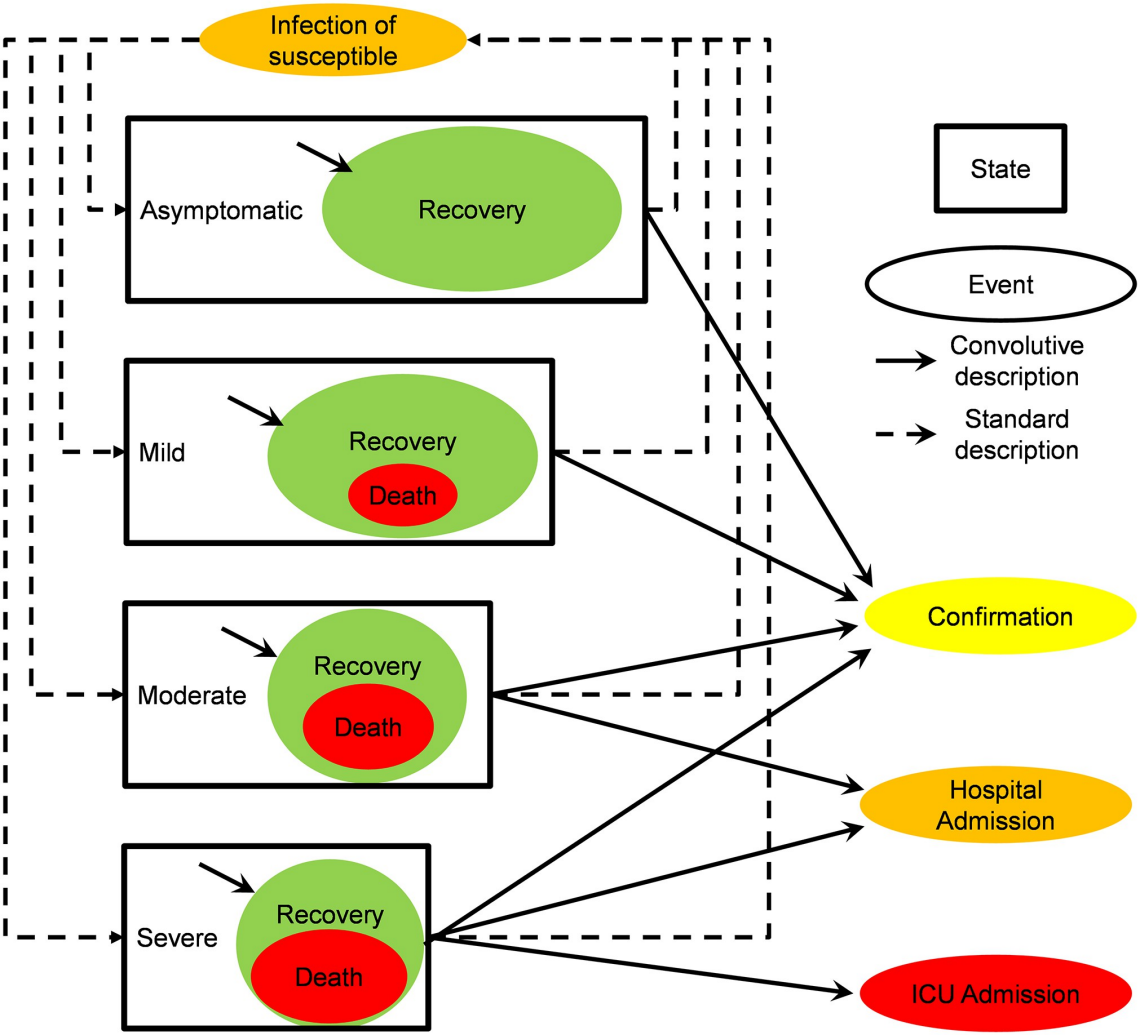
Disease states, events and transitions. We treat all the infected states (asymptomatic, mild, moderate and severe) as a source of recovered or dead individuals, confirmed cases, and hospital and ICU admissions. The infectious individuals in each case are the difference between the infected and the recovered or dead ones for each case. The four infected types trigger events by means of both, standard rates (dashed lines) and convolution integrals (solid lines). Thus, confirmed, hospitalised and ICU states are fed by events triggered by the infection date with a different probability of occurrence for each type. The infectious individuals of each type feed the infection event on the susceptible individuals with a different standard rate. The infected individuals of each type feed the recovery or death event with a different median and IQR.

The objective is to replicate the real evolution of the COVID-19 disease in the region of Madrid (*Comunidad de Madrid*, CM). The full region has been considered as one single region (NUTS-2 & NUTS-3), where the population has been firstly classified as susceptible, infected and recovered or dead. This model is more general than the basic model presented in Eq (3) because the infected and the recovered or dead individuals have been secondly classified into four types: asymptomatic, mild, moderate and severe. Fig 1 graphically represents the disease states, events and transmissions, where the arrows represent the temporal evolution of the individuals. There are two types of temporal evolutions: events that have been mathematically modelled by a standard rate term (dashed lines in Fig 1) and events that have been mathematically modelled by a convolution integral (solid lines). Thus, each solid line has its own distribution function, that is, its own median and IQR. Readers can find the final equations, along with a detailed description of the complete model, S1 Text.

The flow in Fig 1 is explained as follows: a susceptible individual becomes infected as a particular type (asymptomatic, mild, moderate or severe) and then recovered or dead. The model requires input of the fraction of each type in the population of infected individuals. Meanwhile such infectious persons can produce: 1) new infections, 2) positive confirmation, 3) hospital admission 4) ICU admission and 5) recovery or death. The death event has been associated to the event of recovery with a different probability for each type; thus, there is a fatality rate for each infected type. Fig 1 represents this fact by including the death inside each recovery box.

All the severe cases will be admitted in the ICU and this event is associated with a C-model (i.e. a convolution integral similar to that shown in Eq 3 and which is fully explained S1 Text) that uses a median and an IQR to determine when they will be admitted. All the severe and the moderate cases will be admitted to the hospital, which uses another C-model to distribute the admissions using a distribution with the median and the IQR of each type. In addition, all the types are exposed to the event of being confirmed as COVID-19 carriers with a different probability distribution for each type (proportion, median and IQR). This fact establishes the confirmed cases as another C-model. Finally, the proposed description leads to a model with 56 parameters. Table 1 collects a listing of all the model parameters and gives a brief description of them.

**Table 1:** List of parameters used in the proposed model. Additional details are in S1 Text.

Agent	Event	Parameter	Description	Range
General population:	Transmission	*P*_*T*_	Total number of individuals	[1, ∞)
		*χ*	Fraction of accessible individuals	[0, 1]
		*ω*	General mobility parameter (Depends on the normal social mobility/activity in absence of disease)	[0, ∞)
		*β*	Particular mobility parameter (Depends on the particular restrictions adopted by the government in the presence of the disease). Sometimes referred to as stringency index [47]	[-1, ∞)
Infected, type *i* ∈ {0, 1, 2, 3,…} 0 = Asymptomatic 1 = Mild 2 = Moderate 3 = Severe	Transmission	*α*_*i*_	Particular mobility parameter (Depends on the severity of the disease: severe infections have a less value than asymptomatic ones)	[0, 1]
		*γ*_*i*_	Probability of success in transmitting the virus from the infectious to the susceptible	[0, 1]
		*ϕ*_*i*_	Probability of being infected as type *i*	$\left[0,1\right]$ $\sum_{i}\phi_{i}=1$
	Recovery or death	*Median*_*i*_	Median and interquartile range of the probability distribution for the recovery or death event of an infected of type *i*	(0,∞)
		*IQR*_*i*_		(0,∞)
	Death	${\psi_{D}}_{i}$	Fatality rate for an infected of type *i*	[0, 1]
	Hospital admission	*Median*_*i*_	Median and interquartile range of the probability distribution for the hospital-admission event of an infected of type *i*	(0,∞)
		*IQR*_*i*_		(0,∞)
	ICU admission	*Median*_*i*_	Median and interquartile range of the probability distribution for the ICU-admission event of an infected of type *i*	(0,∞)
		*IQR*_*i*_		(0,∞)
	Confirmation	${\chi_{P}}_{i}$	Fraction of infected of type *i* who are detected by the screening tests	[0, 1]
		*Median*_*i*_	Median and interquartile range of the probability distribution for the confirmation-as-positive event of an infected of type *i*	(0,∞)
		*IQR*_*i*_		(0,∞)

### 2.4 Ethical considerations

Data has been obtained from the Epidemiological Surveillance Network and therefore, no explicit ethical evaluation is necessary.

### 2.5 Data

Five time series have been used on a daily basis: confirmed, asymptomatic, hospital, ICU and deaths. Data was obtained from the Public Health Information and Alerts System (SISPAL) where notifications of suspected, probable and confirmed cases of COVID-19 were registered to the Epidemiological Surveillance Network of the *Comunidad de Madrid* (CM). Only confirmed cases are included in the analysis according to the case definition available in [48]. The confirmed cases are automatically captured from the positive diagnostic tests sent by the CM’s public and private laboratories. The case assignment date corresponds to the laboratory date and, failing that, to the date of onset of symptoms, admission or notification. Hospital and ICU admission and discharge dates are automatically captured from hospital information. The date of death is assigned through a daily notification of the deceased in public and private hospitals and retrospectively through cross-checks with the database of funeral services. Classification as an asymptomatic case is carried out through the epidemiological survey.

**Fig 2:**
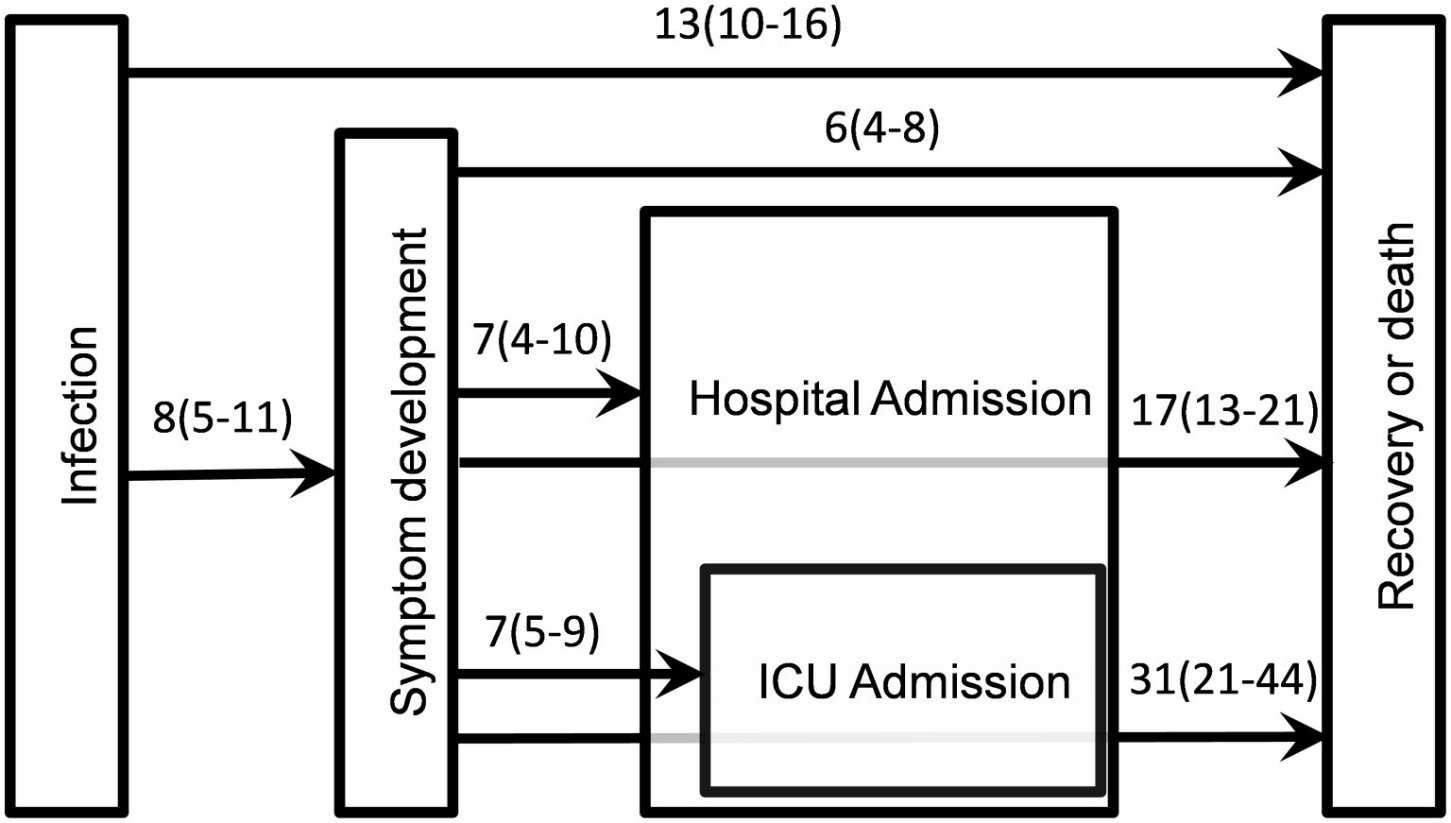
Medians and Interquartile Ranges (IQR) for the main disease events. Medians and IQRs for hospital and ICU admissions and recoveries have been obtained from SENTINEL and are measured in days elapsed from the onset of symptoms, which is assumed to coincide, on average, with the confirmation date.

The Sentinel Surveillance System of Severe Acute Respiratory Infection (SENTINEL) collects data on hospitalized cases from three acute care hospitals in Madrid covering 1528097 inhabitants. This system provides clinical information on laboratory-confirmed SARS-Cov-2 patients and is updated weekly. These data allow us to know fatality rates as well as medians and IQRs for admissions and discharges, which are collected in Fig 2.

The medians and the IQRs obtained from the data reported by hospitals (SENTINEL) give the periods from the onset of symptoms and hence there exists a delay since the infection takes place. This delay has been estimated to be in the range (2–14) days which allows us to fix the median and the IQR respectively as 8 and (5–11) for the initial period of Fig 2. Assuming that consecutive events can be added up, the infection event is set as the trigger signal. In addition, the particular mobility parameter, *α*_*i*_, can be estimated taking into account that mobility is highly reduced once the symptoms appears. For this reason, it is plausible to assume that *α*_*i*_ = MedianToSymptoms_*i*_/MedianToRecovery_*i*_ holds. These numbers will be used as the input fixed parameters of the model and are collected in Table 2.

**Table 2:** Values used in the C-model for the fixed input parameters.

Event	Parameter	Value	Source
Transmission	*P*_*T*_	6.8 10^6^	Population of Madrid at the initial date.
	*χ*	1.0	Assumption: all the population is accessible.
	*ω*	5	Assumption: plausible value.
Transmission	*α*_0_	1.000	Calculated as
	*α*_1_	0.571	MedianToSymptoms_*i*_/MedianToRecovery_*i*_
	*α*_2_	0.320	See discussion in the text.
	*α*_3_	0.258	
Recovery or death	*Median*_0_	13 days	SENTINEL
	*Median*_1_	14 days	
	*Median*_2_	25 days	
	*Median*_3_	39 days	
	*IQR*_0_	6.0 days	SENTINEL
	*IQR*_1_	7.2 days	
	*IQR*_2_	10.0 days	
	*IQR*_3_	23.8 days	
Death	${\psi_{D}}_{0}$	0.000	SENTINEL
	${\psi_{D}}_{1}$	0.000	
	${\psi_{D}}_{2}$	0.170	
	${\psi_{D}}_{3}$	0.279	
Hospital admission (types 2 to 3)	*Median*	15 days	SENTINEL
	*IQR*	8.5 days	SENTINEL
ICU admission (type 3)	*Median*	15 days	SENTINEL
	*IQR*	7.2 days	SENTINEL
Confirmation (types 0 to 3)	${\chi_{P}}_{2}$	1.000	Assumption: all the cases are tested.
	${\chi_{P}}_{3}$	1.000	
	*Median*	8 days	SENTINEL
	*IQR*	6.0 days	SENTINEL

**Fig 3:**
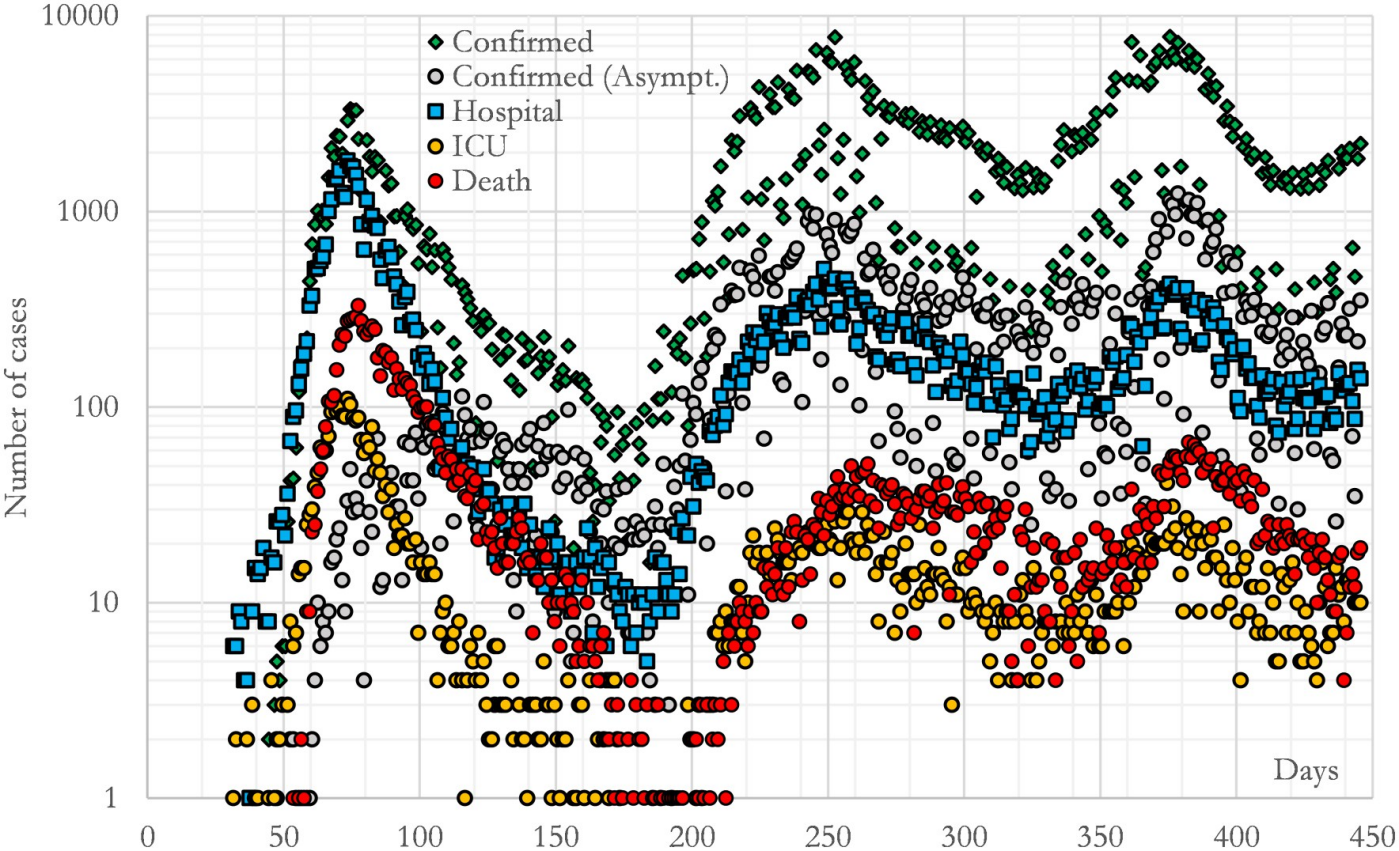
Evolution of confirmed, asymptomatic, hospitalized, ICU and death cases during the studied period in the region of Madrid (CM). Data come from SISPAL as explained in the text. The main dispersion in the data comes from the delay in reporting cases during the weekend. This effect moves some Saturday and Sunday cases towards Monday.

Fig 3 shows the official daily evolutions reported by SISPAL of the selected series during the first three waves in the region of Madrid (CM). The initial date reported was 10^th^ February 2020 with 6 hospital admissions and 1 ICU admission. The final date was 28^th^ March 2021. We have selected this final date in order to have the longest period with a negligible number of vaccinated individuals. In addition, this period comprises three waves, which gives us the ability to assess the behaviour of the model under different social conditions.

The dispersion observed in the time series of Fig 3 comes from a recurrent shift during the weekends (some of the Saturday and Sunday cases were assigned to Monday due to the way in which some cases were communicated to the authorities). This delay was more severe for asymptomatic and mild cases since they were detected outside hospitals. The result is a broadening of the curve for such time series. We have preferred not to artificially remove this dispersion.

The logarithmic axis in Fig 3 represents the number of cases per day, where it can be seen that the peak was 1833 hospital admissions in the same day, this happened 52 days after the first admissions were reported. Something similar occurred with the critical-care cases, which had a peak of 110 admissions (also 52 days after the first hospital admission was reported).

It is interesting to note that during the first wave (before 28^th^ June 2020) only the hospital and ICU admissions can be considered as “well-known”. This is because the hospital underwent tests on all the patients who were susceptible to the COVID-19 disease whereas outside hospitals there was not a generalized testing procedure (by 6^th^ August less than 10% of the Madrid’s population had been tested [4–6]). Thus, it is plausible that confirmed and asymptomatic individuals were significantly underreported during the first wave due to a lack of screening tests. Estimating this number is one of the purposes of this study.

The curve of deaths is also remarkable: the number of cases, which are well identified and documented, indicates a higher fatality rate during the first wave (during this period there were no previous experience and protocols). Estimating the excess of fatality during these initial days is another objective of this work. In contrast, we consider that the five time series do not present the previous bias from 28^th^ June 2020 to 28^th^ March 2021 (second and third waves) because for that time, testing was being massively done to different agents in the population (teachers, health personnel, etc.) as well as all suspicious cases and close contacts.

### 2.6 Model fitting

The mathematical model was written in an Excel workbook and fitted with the built-in Excel’s solver (see S1 Data) using a significant number of initial seeds. The procedure was as follows. Step 1: All the parameters which are labelled as “Fixed” in Table 3 were kept constant during the optimization process. Step 2: All the parameters which are labelled as “Fitted” in Table 3 were initially filled with a plausible guess value. Step 3: The full mathematical model was integrated from the initial time to the final time in order to obtain the time series. Step 4: The differences between the calculated and empirical time series were used to calculate a global error for the calculation. Step 5: A Non-linear Central-Derivatives Generalized Reduced Gradient optimizer (a built-in option in Microsoft Excel) was used to find a new point with a lower global error. (During this step, the parameters labelled as “Fitted” in Table 3 were changed). Step 7: The procedure was repeated while the partial derivatives were not zero (the convergence parameter was selected as 10^−7^). Step 8: Once a solution was found, a new starting seed was proposed and the procedure was restarted from step 3. If the found error was larger than the minimum already achieved, the solution was rejected.

**Table 3:** Values of all the parameters used in the C-model.

Agent	Event	Parameter	Value	Source
All	Initial time		10/01/2020 9:35:44	**Fitted**
General population:	Transmission	*P*_*T*_	6.8 10^6^	Fixed
		*χ*	1.0	Fixed
		*ω*	5	Fixed
		*β*	See Fig 9	**Fitted**
Infected, type *i* ∈ {0, 1, 2, 3, …} 0 = Asymptomatic 1 = Mild 2 = Moderate 3 = Severe	Transmission	*α*_0_	1.000	Fixed
		*α*_1_	0.571	Fixed
		*α*_2_	0.320	Fixed
		*α*_3_	0.258	Fixed
		*γ*_0_	0.0670	**Fitted**
		*γ*_1_	0.0693	**Fitted**
		*γ*_2_	0.0693	**Fitted**
		*γ*_3_	0.0694	**Fitted**
		*ϕ*_0_	0.2761	**Fitted**
		*ϕ*_1_	0.6766	**Fitted**
		*ϕ*_2_	0.0439	**Fitted**
		*ϕ*_3_	0.0034	**Fitted**
	Recovery or death	*Median*_0_	13 days	Fixed
		*Median*_1_	14 days	Fixed
		*Median*_2_	25 days	Fixed
		*Median*_3_	39 days	Fixed
		*IQR*_0_	6.0 days	Fixed
		*IQR*_1_	7.2 days	Fixed
		*IQR*_2_	10.0 days	Fixed
		*IQR*_3_	23.8 days	Fixed
	Death	${\psi_{D}}_{0}$	0.000	Fixed
		${\psi_{D}}_{1}$	0.000	Fixed
		${\psi_{D}}_{2}$	0.170	Fixed
		${\psi_{D}}_{3}$	0.279	Fixed
	Hospital admission (types 2 to 3)	*Median*	15 days	Fixed
		*IQR*	8.5 days	Fixed
	ICU admission type 3)	*Median*	15 days	Fixed
		*IQR*	7.2 days	Fixed
	Confirmation (types 0 to 3)	${\chi_{P}}_{0}$	0.286	**Fitted**
		${\chi_{P}}_{1}$	0.650	**Fitted**
		${\chi_{P}}_{2}$	1.000	Fixed
		${\chi_{P}}_{3}$	1.000	Fixed
		*Median*	8 days	Fixed
		*IQR*	6.0 days	Fixed

This procedure fits the model to the five time series previously described: confirmed, asymptomatic, hospitalised, ICU and deaths. As mentioned before, we consider that the five curves are valid for calculating the global error from 28 June 2020 onwards and that only hospital and ICU admissions are valid before. Although the objective is to fit the curves in a daily basis we have also used the cumulative hospital and ICU admissions to calculate the global error. This way, the calculated final number of cases in each curve can be considered as a proxy of the real number, which is unknown (for the reasons explained in the previous section) for confirmed and asymptomatic cases. This is also useful to estimate how many deaths were due to a lack of COVID-19 specific procedures during the first wave.

The model has 56 available parameters but only 12 of them have been determined by minimizing the error. We have selected these parameters to avoid the redundancy between the parameters. In this way, the parameters which would not be distinguishable during the model fitting do not lead to identifiability issues. In general, all these fitted parameters could be functions of time, but only the parameter *β* (similar to the stringency index [47]) was allowed to change with time. This way, the model has a unique parameter to take into account the real social interaction (a mixture of mobility, social distancing, mask effects, etc.). To keep the computational cost under control, the parameter *β* has been treated as a piecewise function with 98 sections, with each section beginning and ending on a socially significant date (see Fig 9). Therefore, the model has 110 degrees of freedom to be adjusted.

The initial time of the propagation has a significant influence in the resulting time series and it is completely unknown; hence, it is one of the free parameters whose value has to be obtained by minimizing the error. The obtained value is 10^th^ January 2020 9:35. In contrast, the final date used in the calculus is fixed to 28^th^ March 2021 23:59. The number of steps between both dates is fixed to 2636, what means that the time step changes during the minimization of the error. Its final value was 0.1687 days. The main reason to keep the number of steps low (2636 in any case) is the required computational time. Note that the model has to solve 12 differential equations for each set of parameters, which would not represent a significant problem if the proposed model did not include so many convolution integrals. In particular, the model studied here requires 7 convolution integrals which must be completely recalculated at each integration step. This makes the execution time in the order of minutes when the number of steps is in the range of 2500. Since the optimization process requires numerically evaluating the gradient, the complete model has to be executed 110 times each time the optimizer wants to advance towards the minimum. Taking into account that the model requires nearly 100 optimization steps to achieve an acceptable zero gradient, the time necessary to adjust the values is tens of thousands of minutes, that is, approximately a week. Thus, the calculation time is near a week per each new seed used as a starting point. Table 3 collects both, the fixed parameters used and the fitted parameters obtained.

## 3. Results

**Fig 4:**
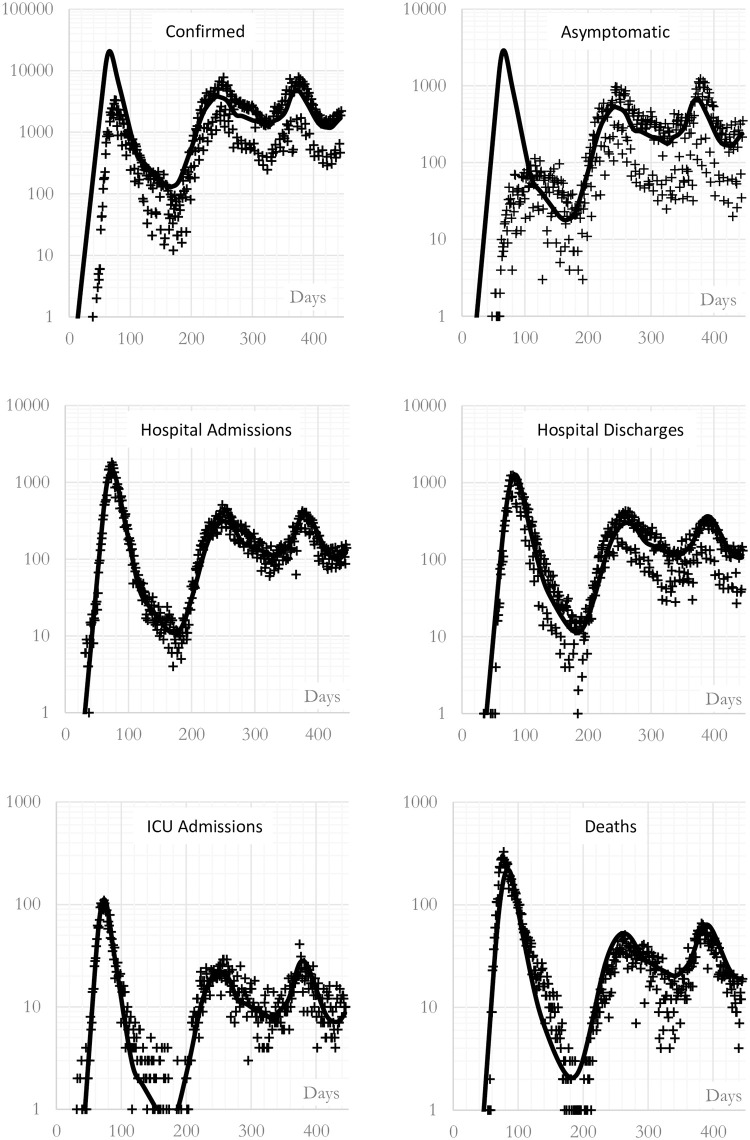
Calculated evolution of confirmed, asymptomatic, hospitalized, ICU and death cases during the studied period in the region of Madrid (CM). Lines are the curves calculated with the convolution mathematical model presented in this study. Points are actual data (SISPAL).

The process described in the previous section leads to the curves presented in Fig 4, where the officially reported points and the calculated lines are simultaneously drawn. The proposed fitting process ensures that 1) the calculated number of hospitalised and ICU daily cases are in agreement with the empirical data during the three waves, 2) the confirmed (total), asymptomatic (confirmed) and death daily cases are in agreement with the empirical data only during the second and third waves, and 3) the officially confirmed (total), asymptomatic (confirmed) and death daily cases during the first wave are not communicated to the model. This has been done on purpose precisely to determine this missing information during the first wave. This way, the differences between the calculation and the reported values appear clearly when comparing the cumulative values at the end of the first wave and at the end of the studied period, which are respectively presented in Table 4 for the first wave and Table 5 for the other two waves. The interpretation of these tables requires knowing two of the fitted parameters in Table 3, precisely, *ϕ*_0_ = 0.276 and ${\chi_{P}}_{0}=0.286$, which estimate the total number of asymptomatic infections near 30% of the total infections and the confirmed asymptomatic near 30% of the asymptomatic individuals.

**Table 4:** Cumulative cases at the end of the first wave (28^th^ June 2020).

Curve	Epidemiological Surveillance data	Calculated	Difference	Difference respect calculated (%)
Confirmed	76230	449491	-373261	-83.0%
Asymptomatic (confirmed)	4560	62667	-58107	-92.7%
Hospitalised (confirmed)	37093	37482	-389	-1.0
ICU (confirmed)	2671	2670	1	0.0
Death (confirmed)	8674	6637	2037	30.7
Infected (total)	Not Available	796143		
Recovered (total)	Not Available	792725		

**Table 5:** Cumulative cases from the start of the second wave to the end of the third wave (from 28^th^ June 2020 to 28^th^ March 2021).

Curve	Epidemiological Surveillance data	Calculated	Difference	Difference respect calculated (%)
Confirmed	558382	547666	10716	2.0%
Asymptomatic (confirmed)	76164	76354	-190	-0.2%
Hospitalised (confirmed)	46852	44976	1876	4.2%
ICU (confirmed)	3207	3204	3	0.1%
Death (confirmed)	5984	7794	-1810	-23.2%
Infected (total)	Not Available	991990		
Recovered (total)	Not Available	952485		

The relative difference at the end of the studied period in all the waves is less than 5% for the hospitalised cases and almost zero for the ICU cases. For the second and third waves, the error is less than 2% for the confirmed cases, including the asymptomatic ones. These low values for these errors ensure that the model is correctly calculating the final cumulative number and that can be used to estimate the missing information. Thus, as expected in the first wave, these two last differences increase dramatically to respectively -83% (confirmed) and -92.7% (asymptomatic), showing the magnitude of the lack of testing during the first wave (there were not enough tests to screen all the population who were outside the hospitals). It is remarkable that the model predicts an underestimation of asymptomatic individuals during the first wave, near 93%, just as it was advanced in the introduction as a plausible explanation of the virulent outbreak.

In addition, the model underestimates the fatality rate during the first wave by 30% and overestimates the fatality rate during the second and third waves by -23%, but both effects compensate each other and the final error for the three waves is inferior to 2%, ensuring again that the calculation of the final cumulative number yields the measured value. More precisely, Tables 4 and 5 show that the cases reported as hospital and ICU admissions at the end of the first wave and during the second and third waves coincide with the calculated ones (the errors are -1.0%, 0.0%, 4.2% and 0.1%), whereas the deaths reported do not coincide with the calculated ones at the end of the first wave and during the second and third waves (the errors are 30.7% and -23.2%); however, they do coincide at the end of the third wave (8674+5984 = 14658 measured deaths and 6637+7794 = 14431 calculated deaths, which means an error of -1.5%). This means that, as said, the calculation underestimates the deaths in the first wave and overestimates the deaths during the second and third waves. As can be seen in Table 3 the fatality rates used by the model are fixed at 17.0% for the moderate cases and at 27.9% for the severe cases. Therefore, the difference between the calculated and measured cases suffers a 53.9% change between the first wave and the others. As long as the calculation has been made with a constant fatality rate, we can conclude that the first wave was significantly more lethal than the following ones. As long as the cumulative final number is correct, the conclusion that the fatality rate was not constant is also correct. This indicates that future works related to the accurate simulation of deaths during this initial wave should let fatality rates change over time.

**Fig 5:**
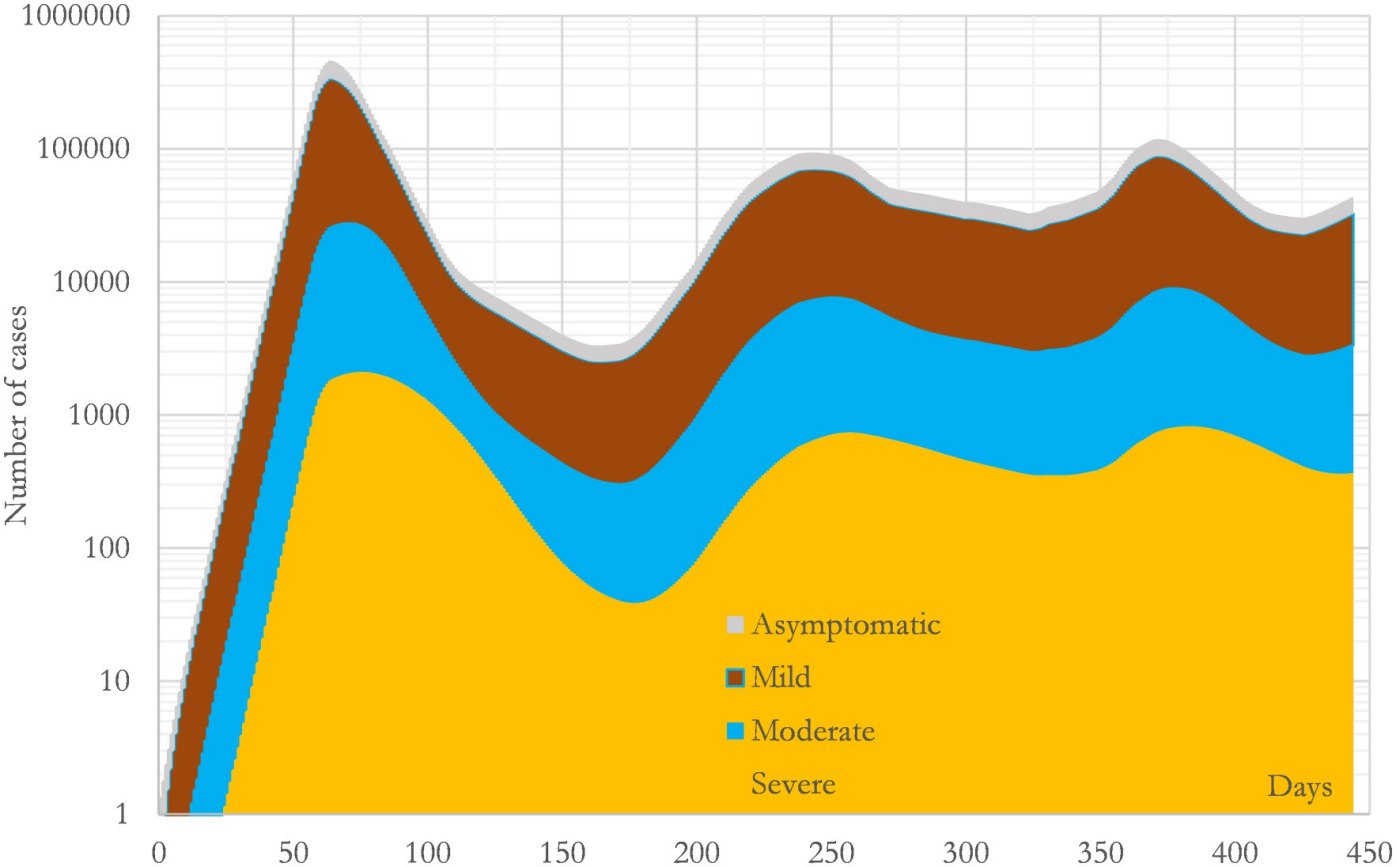
Calculated evolution of the daily prevalence during the studied period in the region of Madrid (CM). The curves present the number of infectious individuals who existed each day segregated by types. All the curves have been calculated with the convolution mathematical model presented in this study using the parameters collected in Table 3.

**Fig 6:**
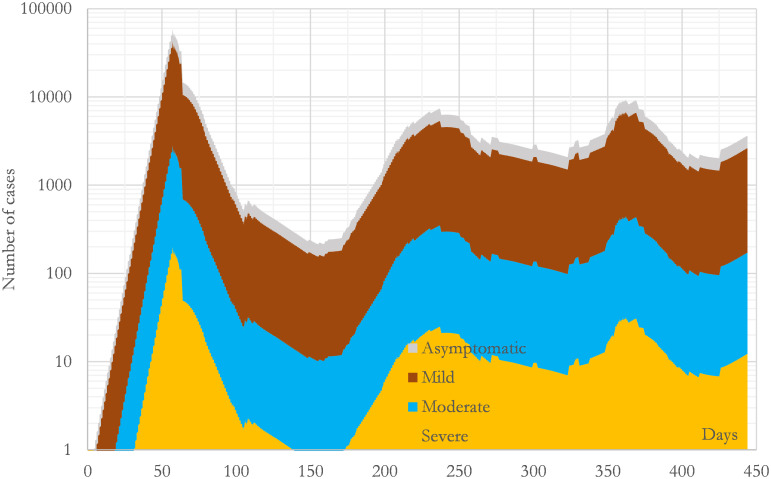
Calculated evolution of the incidence during the studied period in the region of Madrid (CM). The curves present the number of new daily infections which existed each day segregated by types. All the curves have been calculated with the convolution mathematical model presented in this study using the parameters collected in Table 3.

One of the advantages of the mathematical model is that it can provide an estimation of variables which are extraordinarily complex to obtain in the real field. For example, the total number of infectious individuals or the total number of daily infections. Figs 5 and 6 show respectively the evolution of both time series. The curves in Figs 5 and 6 are segregated by types of infection showing that the largest group was the mild one, followed respectively by the asymptomatic, moderate and severe groups. This can be done because the infected and recovered individuals who were presented in the population are a direct result of the calculation, which includes the number of asymptomatic individuals. In particular, Fig 5 states that during the first wave the peak happened after 64 days from the onset and presented 124604, 311913, 23636 and 1836 infectious individuals of asymptomatic, mild, moderate and severe types respectively. The calculated (fitted) relative presence of each type appears in Table 3: asymptomatic 27.6%, mild 67.7%, moderate 4.4% and severe 0.3%.

**Fig 7:**
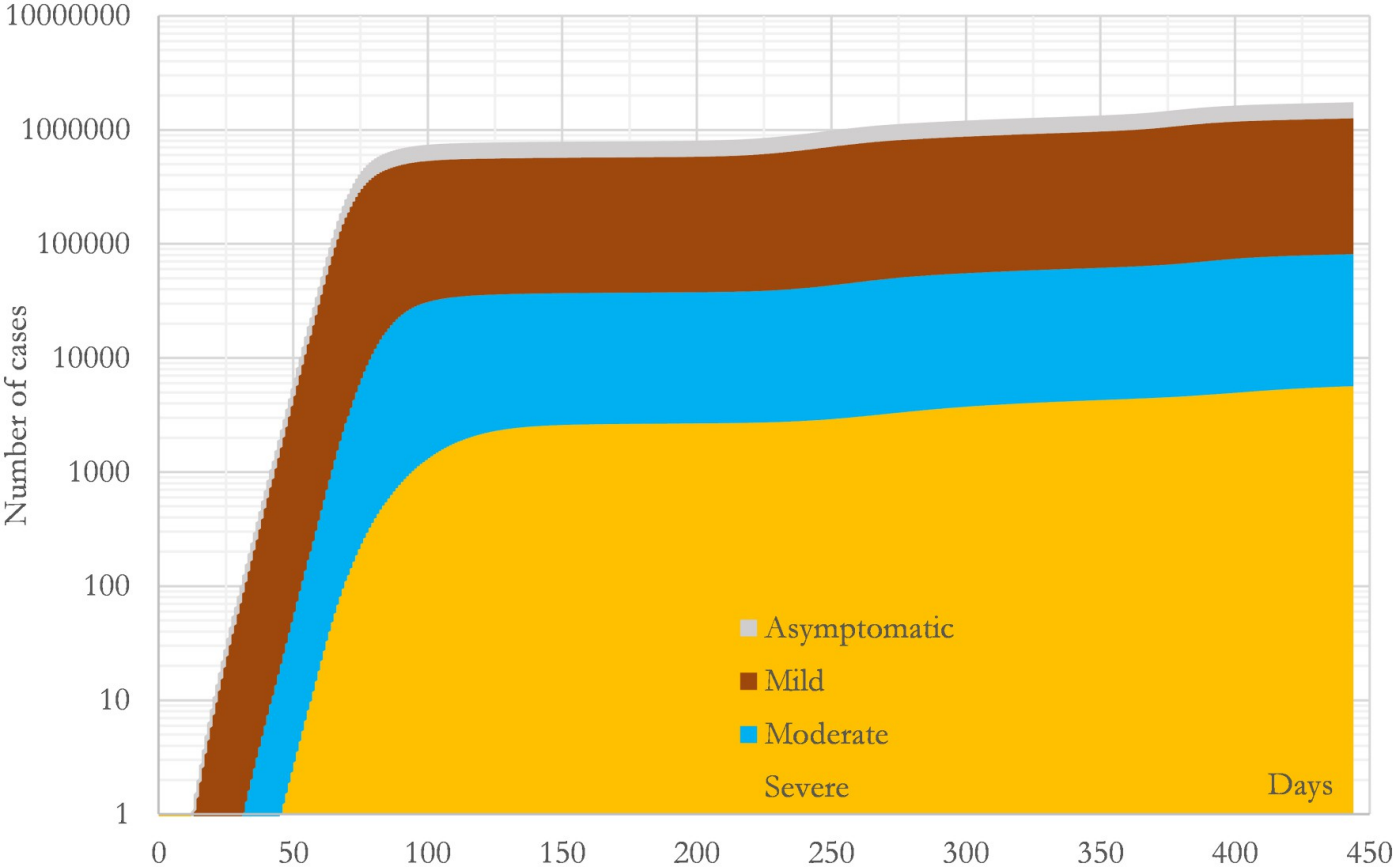
Calculated evolution of the accumulated prevalence during the studied period in the region of Madrid (CM). The curves are cumulative and present the number of recovered individuals segregated by types. All the curves have been calculated with the convolution mathematical model presented in this study using the parameters collected in Table 3.

Another interesting question that the model addresses, which is difficult to obtain by a direct measurement, is the number of individuals who have recovered or died, and hence, cannot propagate the virus anymore. (Here we are assuming that all the recovered individuals who are still alive have a high probability of having developed a strong and permanent immunity to the virus.) This evolution is presented in Fig 7 segregated by types. By the end of the studied period the number of recovered individuals from each type, asymptomatic, mild, moderate and severe, were respectively 483389, 1182678, 75683 and 5666. In this case, the estimation is that by the end of the period there were 1747418 recovered individuals. This is an important number since it measures the degree of dissemination: at the end of the third wave the model states that 26% of the CM’s population had developed immunity.

**Fig 8:**
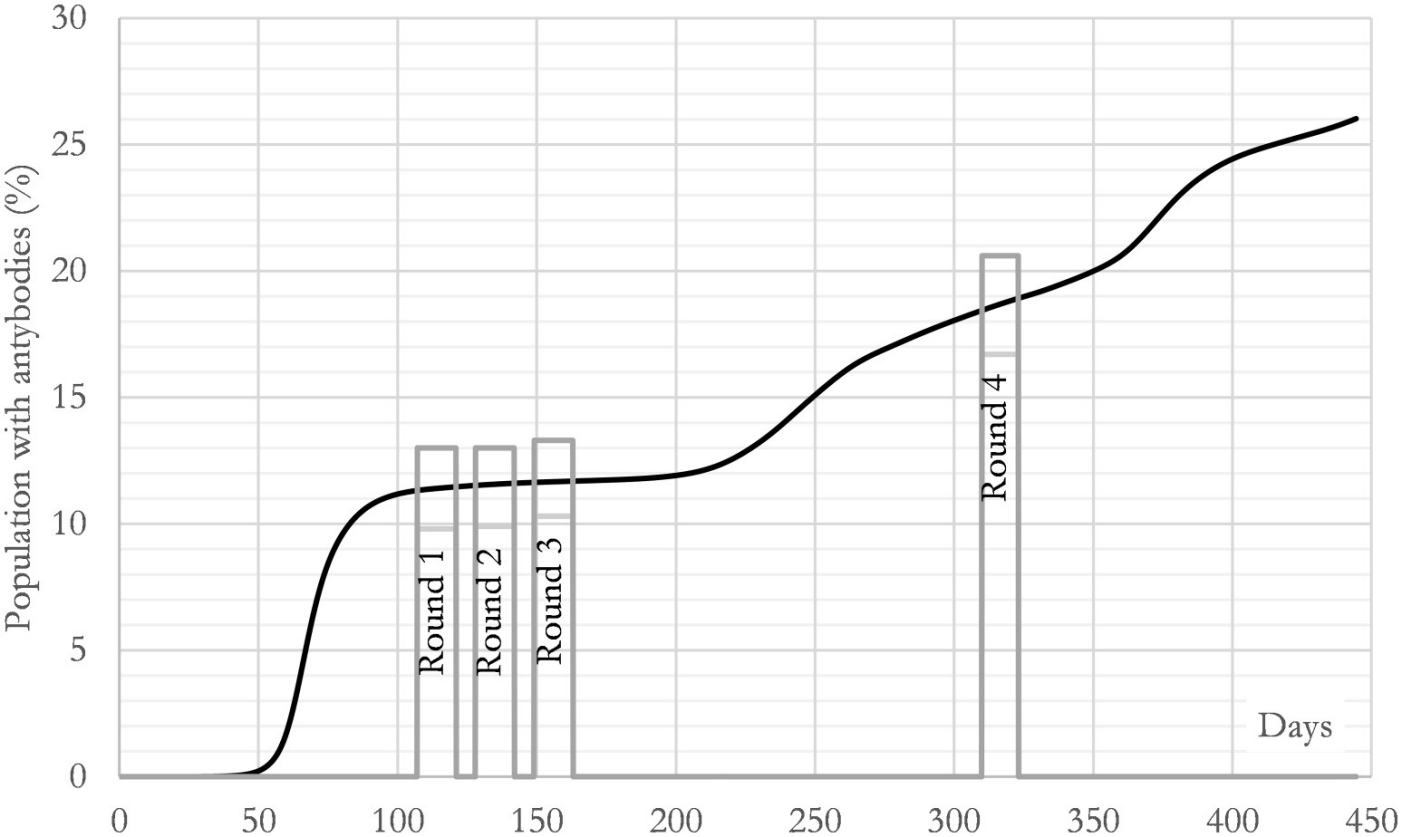
Calculated evolution of the infected individuals as a percentage of the total population in the region of Madrid (CM). Bars are the 95% confidence intervals in each round conducted by the ENE-COVID research [49, 50], which gives a measure of the persons with IgG antibodies against SARS-CoV-2. The solid line is the calculation made with the proposed C-model.

These results are compatible with the ENE-COVID study, a sero-epidemiological research for the infection by SARS-CoV-2 in Spain [49–52]. The study was conducted in all the regions of Spain and comprised four temporal windows: Apr 27-May 11 (round 1), May 18-Jun 1 (round 2), Jun 8-Jun 22 (round 3) and Nov 16-Nov 29 (round 4). In particular, in the fourth round, the percentage of people with IgG antibodies against SARS-CoV-2 in the region of Madrid was close to 18.6% (95%CI: 16.7–20.6). Fig 8 shows the curve calculated in this article (solid line) and the 95% confidence intervals obtained in the ENE-COVID research for each round. The ENE-COVID study gave the following 95% confidence intervals for the four rounds: round 1 [9.8, 13], round 2 [9.9, 13], round 3 [10.3, 13.3] and round 4 [16.7, 20.6]. The calculation gives the following evolution during each round: round 1 [11.3, 11.5], round 2 [11.5, 11.6], round 3 [11.6, 11.7] and round 4 [18.4, 18.9]. This means that the centres of the intervals reported by the ENE-COVID study and the centres calculated in this article differ less than 1.5% for all the rounds. As it can be seen, the agreement between both studies is high.

**Fig 9:**
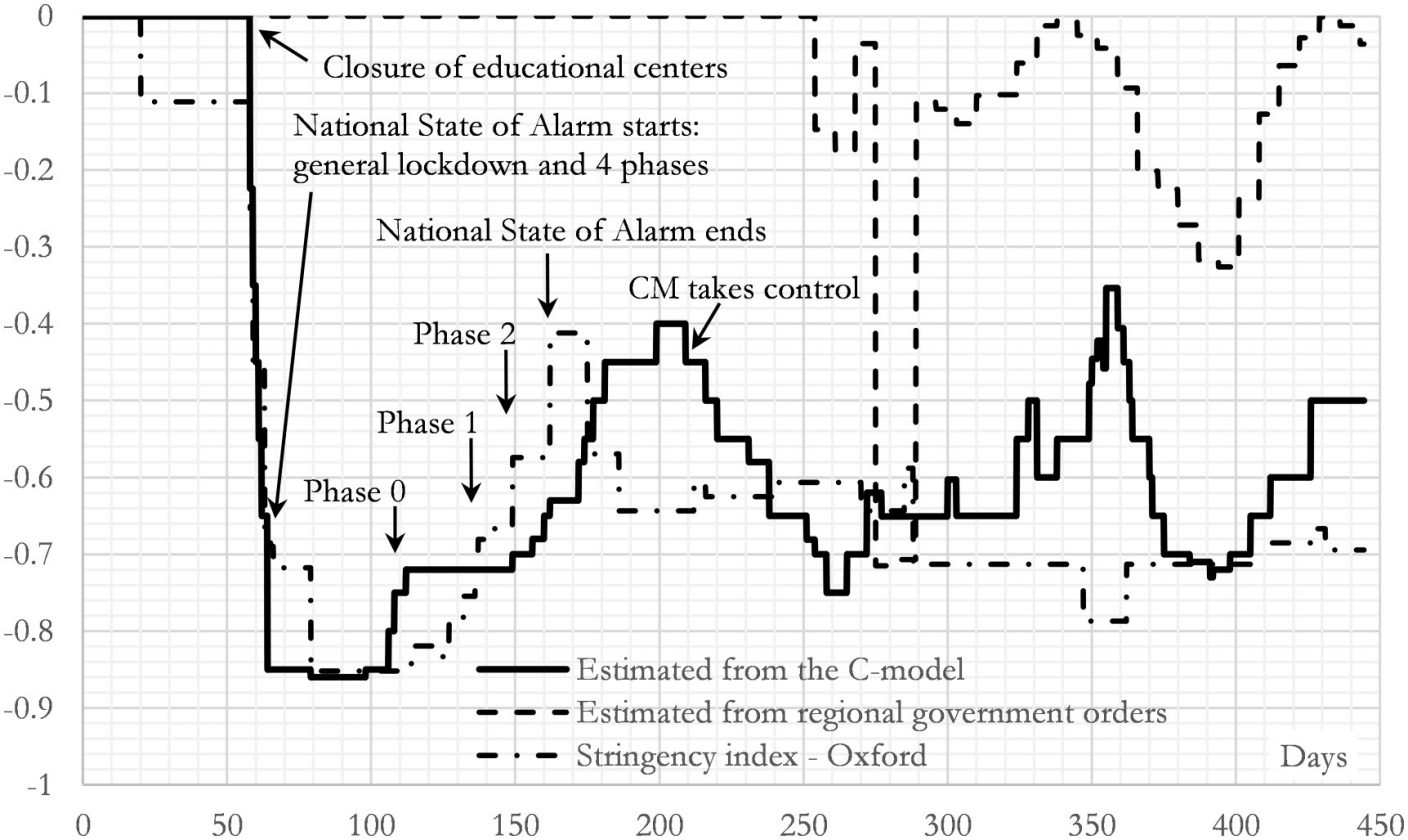
Estimation of the mobility parameter (Stringency index) during the studied period in the region of Madrid (CM). The solid line is the curve obtained by fitting the C-model: it ensures that the C-model generates the official time series shown in Fig 3. The lower dash line shows the estimation of the Stringency Index of Spain made by [47] taking into account the national restrictions. The upper dash line shows an estimation of the CM’s population affected by the local government restrictions (without taking into account the national restrictions).

Finally, another interesting result of this study is the indirect measurement of the parameter *β*, which, in this model, is a measure of how effective the different restrictions imposed by the government have been. We have defined mobility in such a way that the mobility parameter *β* is zero when society is behaving normally. It is greater than zero if there is more interactivity than normal and less than zero when interactivity is less than normal. In particular, a value of -1 means that any person-to-person transmission is completely avoided. This means that any governmental action focused on slowing the social interactions will lead to *β* ∈ [–1, 0]. As long as in this work *β* has been considered as a piecewise function of time with 98 relevant dates distributed according to relevant legislative dates, we have 98 values of *β* along such period. These 98 values have been obtained by fitting the model to the official time series and hence they are a result of the model. These calculated values of *β* are drawn (solid line) as a function of time in Fig 9, which shows that β has a value of 0 at the beginning of the breakout in Madrid and becomes less than 0 when the different restrictions are being imposed.

Fig 9 also compares this mobility parameter with two others. The first one is the stringent index developed by the Oxford University [47], who published the value of such index for Spain. As can be seen in the figure, there is a good correlation between the β factor and the Oxford Stringency index almost during the first 150–200 days. After this period the correlation is lost. The explanation is that the national government transferred the control of the restrictions to the regional government on 21^st^ June 2020. Therefore, the second one is the fraction of population which is under a local closure imposed mainly by the regional government (imposing several local mobility restrictions in different basic health zones in Madrid [6]), not by the national one. As can be seen in Fig 9, this second source is a partial explanation of the found behaviour. Indeed, as expected, the real behaviour seems to be a mixture of both, the regional and the national restrictive measures. Thus, the three different measures are comparable and are somehow correlated: the calculated one is a mixture of the other two.

In the figure, it is also possible to identify some of the most important events of government policy. For example, during the first year of pandemic in Spain, the National Government decreed three States of Alarm with different degrees of mobility along them. In addition, since 21^st^ June 2020, the Madrid’s Regional Government (CM) has had additional autonomy to tighten or weaken the mobility restrictions, which in fact also affected the pandemic evolution in the region. Thus, the parameter *β* could be a good proxy of this stringency index (containment and closure policies, sometimes referred to as lockdown policies) [47].

## 4. Discussion

The mathematical model has been built using different layers. Firstly, we have implemented a compartmental model of type SI^4^R^4^ (the superscript indicates that it uses four types of infected and recovered individuals). Secondly, we have completed the model with three additional events leading to a SI^4^R^4^H^2^U^1^D^4^ model (the new superscripts indicate that Hospitalised comes from moderate and severe, ICU comes from severe, and Death comes from all of them). Finally, the use of convolution integrals to distribute the events over the time span leads to the C-I^4^R^4^H^2^U^1^D^4^ model that we have used for this study. Up to the authors’ knowledge, such kind of model is new. For this reason, one of the objectives of this study was to assess whether the C-I^4^R^4^H^2^U^1^D^4^ model was capable of correctly dealing with complex situations like the one presented in the worldwide spread of COVID-19. In this quest, we have restricted our analysis to a small (but very complex) area in Spain, the region of Madrid (CM) with nearly 6.8 million people, 37 public hospitals and 286 basic health zones. We think that the result has been satisfactory as the previous section has shown.

The C-I^4^R^4^H^2^U^1^D^4^ model has allowed us to use exactly the same medians and IQRs for the whole period of time, that is, for the three waves. This shows that, as expected, the parameters used to model the health events are independent of time whereas the social events are not. This evidences that the model has the nice-to-have property discussed in the introduction: to keep its input parameters as independent as possible. In particular, the input parameters associated with the health events (recovery, admission in health units, etc.) required to reproduce the measured data for each type of infection are the same regardless of the value that the mobility parameter had, that is, regardless of the rate of activity in the society. But, furthermore, if we examine the Table 3 and count the number of parameters whose value does not change during all the waves, we find they are all of them except the mobility parameter *β*: keeping the number of decoupled parameters as high as possible. Thus, the C-I^4^R^4^H^2^U^1^D^4^ model is expected to be more flexible and robust. This allows the users 1) to describe and calculate the propagation of the illness in complex scenarios, 2) to know the meaning of all the input parameters, and 3) to extrapolate such input parameters (normally, probability distributions of events) towards the future or the past.

In addition, the C-I^4^R^4^H^2^U^1^D^4^ model has another powerful advantage when it is compared with a non-convolutional model. This advantage comes from the integral in the third equation of (3), which tends to smooth out the irregularities provoked by the second equation. (Integrals tend to decrease the noise while derivatives tend to increase it because integrals are numerically calculated as averages multiplied by small numbers whereas derivatives are numerically calculated as differences divided by small numbers.) This fact can be seen in Figs 5 and 6 where it is clear that Fig 5 has removed the irregularities which Fig 6 presents. This mathematical particularity dramatically increases the robustness of the predictions made by the model and helps to deal with uncertainty. Or the other way round, increases the confidence on the fitted parameters found with this model. Obviously, this characteristic is not compulsory, but together with the independence of the parameters, is another nice-to-have feature which helps when we are dealing with machine learning algorithms or fitting models in complex scenarios like the ones provoked by the SARS-CoV-2 all around the world.

However, a drawback of the C-I^4^R^4^H^2^U^1^D^4^ model is that it is no longer a set of ordinary differential equations since, in general, the convolution integral cannot be removed from the system. Therefore, for the same propagation problem, the computational cost increases dramatically. This means that better ways of computing and optimizing must be taken into account. In addition, the model should be adapted to implement additional events such as reinfections and immunity due to vaccination. The inclusion of these new events will require new convolution integrals and hence will increase even more the computational cost.

Finally, we can see the C-I^4^R^4^H^2^U^1^D^4^ model as a link between a set of input parameters *X*_*A*_ (the fixed parameters in Table 3), a set of input parameters *X*_*B*_ (the fitted parameters in Table 3) and a set of output time series *Y* (the curves in Fig 4). The link between these sets appears when the model is run using the input parameters *X*_*A*_ and *X*_*B*_ to obtain the output time series *Y*. Since the time series obtained are unique if the input parameters are not changed, this is a function, say *F*: (*X*_*A*_, *X*_*B*_) → *Y*. Used this way, the model is predictive. However, in this work the known data are the set *X*_*A*_ and a subset of the time series, say *Y*_*known*_ ⊆ *Y* (which is known because it has been empirically determined), while the unknown are the set *X*_*B*_ and the complementary subset of the time series *Y*_*unknown*_ ⊂ *Y* (which is unknown because it has not been empirically determined). Therefore the model can be rewritten as *F*: (*X*_*A*_, *X*_*B*_) → (*Y*_*known*_, *Y*_*unknown*_), which allows us to write the inverse function *F*^−1^: (*X*_*A*_, *Y*_*known*_) → (*X*_*B*_, *Y*_*unknown*_). Used this way, the model is explanatory; the larger the set *Y*_*known*_, the better the explanation. Two important facts are associated to this description: firstly, the set *X*_*B*_ has been carefully selected to avoid redundancy or degeneracy, that is to ensure that the inverse function *F*^−1^ exists when *Y*_*known*_ = *Y* and *Y*_*unknown*_ = ∅; secondly, given the convolutional nature of *F*, there is not a direct procedure to compute *F*^−1^, which can only be computed by means of an indirect procedure. In this article this indirect procedure is as follows: given the known sets *X*_A_* and *Y*_known_*, choose an arbitrary point *X*_B_*, run *F* to obtain *F*(*X*_A_*, *X*_B_*) and check if the part of *F*(*X*_A_*, *X*_B_*) which is predicting the known data, say *F*(*X*_A_*, *X*_B_*)_*known*_, coincides with *Y*_known_*; if they coincide, we can assume that (*X*_B_*, *Y*_unknown_*) = *F*^−1^(*X*_A_*, *Y*_known_*). For this reason, we require two new ingredients: 1) an error function which measures how different is *Y*_known_* from the known part of *F*(*X*_A_*, *X*_B_*) and 2) a sampling procedure to choose different plausible values of *X*_*B*_ in order to find the *X*_B_* which fulfils *F*(*X*_A_*, *X*_B_*)_*known*_ = *Y*_known_*. As long as finding exactly *X*_B_* involves uncertainties, we assume that the point *X*_*B*_ which leads to the minimum difference between *F*(*X*_A_*, *X*_B_*)_*known*_ and *Y*_known_* is the best guess for *X*_B_*. The expected larger robustness derived from the independence of the variables in the set *X*_*B*_ should minimize the influence of such uncertainty. However, this robustness has the price of a very heavy computation. If the number of seeds and steps required by the optimizer is large, the selection of the optimizer appears as a critical point. Once the capabilities of the model have been stated, the next research should be directed to implement a custom optimizer: the built-in Excel optimizer should be removed in future investigations.

Thus, it is necessary to recognize that we have only checked that in the case of the three waves of Madrid’s propagation, the C-I^4^R^4^H^2^U^1^D^4^ model with a set of plausible input parameters (*X*_A_*, *X*_B_*) produces a solution very similar to the measured one *Y*_known_*. Here, similar means that the difference between *F*(*X*_A_*, *X*_B_*)_*known*_and *Y*_known_* is small: it is implicitly assumed that the smaller the difference, the better the results over *X*_B_* and *Y*_unknown_*. For this reason, during this work a huge amount of initial seeds have been used, and all of them led to optimums worse than the optimum finally selected as the reported solution. It is necessary to recognize that the real values of *X*_*B*_ for the three waves in Madrid are completely unknown, and that this article is just an attempt to obtain them. Are they plausible? Yes they are. How much error do they have? We do not know, but we know that those attempts made to improve the solution did not lead to a better one. The maximum we can transmit is that the presented model along with a plausible set of parameters is able to reproduce the three waves in Madrid. As long as this is true, we can assume that the information *X*_A_* used by the model as an input and the output (*X*_B_*, *Y*_unknown_*) are plausibly near the real value. The reasoning is as follows: if the model has predicted the time series *Y*_known_* (the values of *Y* which had been measured), then it is plausible that the model had also predicted the time series *Y*_unknown_* (the values of *Y* which had not been measured). How it is shown that the solution is plausible? We have checked the results using as *Y*_known_* four direct measurements obtained from independent sero-epidemiological researches and six time series with data coming from an epidemiological surveillance database. The calculated *F*(*X*_A_*, *X*_B_*)_*known*_ lays inside the empirical intervals associated to *Y*_known_*. Therefore, there is no reason to think that the calculations are wrong. As long as the calculation gives the corrected values for the known time-series (they lay in the reported confidence intervals for such measuring), we assume that also gives the corrected values for the unknown time-series.

## Supporting information

S1 Text Document describing the mathematical model. (PDF)

S1 Data Workbook implementing the mathematical model as it has been used in this work. (XLSM)

## References

[1] Zhu N, Zhang D, Wang W, Li X, Yang B, Song J, et al. A Novel Coronavirus from patients with Pneumonia in China, 2019. N Engl J Med. 2020; 382(8):727–733 doi: 10.1056/NEJMoa2001017 31978945 PMC7092803

[2] World Health Organization. Novel Coronavirus (2019-nCoV): situation Report, 1. 2020.

[3] Cereda D, Manica M, Tirani M, Rovida F, Demicheli V, Ajelli M, et al. The early phase of the COVID-19 epidemic in Lombardy, Italy. Epidemics. 2021;37:100528. doi: 10.1016/j.epidem.2021.100528 34814093 PMC8605863

[4] Working group for the surveillance and control of COVID-19 in Spain. The first wave of the COVID-19 pandemic in Spain: characterisation of cases and risk factors for severe outcomes, as at 27 April 2020. Euro Surveill. 2020;25(50) 10.2807/1560-7917.ES.2020.25.50.2001431 PMC7812423 33334400

[5] Equipo COVID-19. RENAVE. CNE. CNM. (ISCIII). Situación de COVID-19 en España a 9 de junio de 2021. 2021

[6] Madrid: Servicio de Epidemiología, Dirección General de Salud Pública. Weekly Epidemiological Report, Community of Madrid. 2021. https://www.comunidad.madrid/servicios/salud/vigilancia-epidemiologica

[7] Mizumoto K, Kagaya K, Zarebski A, Chowell G. Estimating the asymptomatic proportion of coronavirus disease 2019 (COVID-19) cases on board the Diamond Princess cruise ship, Yokohama, Japan, 2020. Euro Surveill. 2020; 25(10) doi: 10.2807/1560-7917.ES.2020.25.10.2000180 32183930 PMC7078829

[8] Lu X, Zhang L, Du H, Zhang J, Li YY, Qu J, et al. SARS-CoV-2 Infection in Children. N Engl J Med. 2020; 382(17):1663–1665. doi: 10.1056/NEJMc2005073 32187458 PMC7121177

[9] Bai Y, Yao L, Wei T, Tian F, Jin DY, Chen L, et al. Presumed Asymptomatic Carrier Transmission of COVID-19. JAMA. 2020;323(14):1406–1407. doi: 10.1001/jama.2020.2565 32083643 PMC7042844

[10] Odagaki T. Analysis of the outbreak of COVID-19 in Japan by SIQR model. Infectious Disease Modelling. 2020; 5:691–698 doi: 10.1016/j.idm.2020.08.013 32935071 PMC7484692

[11] Nishiura H, Linton NM, Akhmetzhanov AR. Serial interval of novel coronavirus (COVID-19) infections. Int J Infect Dis. 2020;93:284–286. doi: 10.1016/j.ijid.2020.02.060 32145466 PMC7128842

[12] Liu J, Wang L, Zhang Q, Yau ST. The dynamical model for COVID-19 with asymptotic analysis and numerical implementations. Appl Math Model. 2021;89:1965–1982. doi: 10.1016/j.apm.2020.07.057 32836696 PMC7414781

[13] García-García D, Vigo MI, Fonfría ES, Herrador Z, Navarro M, Bordehore C. Retrospective methodology to estimate daily infections from deaths (REMEDID) in COVID-19: the Spain case study. Sci Rep. 2021; 11:11274. doi: 10.1038/s41598-021-90051-7 34050198 PMC8163852

[14] Fraser C, Riley S, Anderson RM, Ferguson NM. Factors that make an infectious disease outbreak controllable. Proc. Natl Acad. Sci. 2004; 101(16): 6146–6151 doi: 10.1073/pnas.0307506101 15071187 PMC395937

[15] Benavides EM. Robust predictive model for Carriers, Infections and Recoveries (CIR): predicting death rates for CoVid-19 in Spain. ArXiv[q-bio.PE]. March 31, 2020; 2003.13890v1,

[16] Benavides EM. Robust predictive model for Carriers, Infections and Recoveries (CIR): first update for CoVid-19 in Spain. ArXiv[q-bio.PE]. April 12, 2020; 2004.05639,

[17] Brauer F, Castillo-Chavez C. Mathematical Models in Population Biology and Epidemiology. 2^nd^ Edition, Springer, 2012

[18] Smith D, Moore L, The SIR model for spread of disease- Introduction. Convergence. 2004

[19] Yang C, Wang J. A mathematical model for the novel coronavirus epidemic in Wuhan, China. MBE. 2020; 17(3): 2708. doi: 10.3934/mbe.2020148 32233562 PMC7376496

[20] Wu J-T, Leung K, Leung GM. Nowcasting and forecasting the potential domestic and international spread of the 2019-nCoV outbreak originating in Wuhan, China: a modelling study. The Lancet. 2020; 395(10225):689–697. 10.1016/S0140-6736(20)30260-9 PMC7159271 32014114

[21] Okhuense VA. Mathematical prediction for COVID-19 as a global pandemic. Medrxiv. 2020.

[22] Ming WK, Huang J, Zhang CJ. Breaking down of healthcare system: mathematical modelling for controlling the novel coronavirus (2019-nCoV) outbreak in Wuhan, China. BioRxiv. 2020.

[23] Diekmann O, Heesterbeek H, Britton T. Mathematical Tools for Understanding Infectious Disease Dynamics. Princeton University Press, 2013

[24] Iannelli M, Pugliese A. An Introduction to Mathematical Population Dynamics. Along the trail of Volterra and Lotka. Springer, 2014

[25] Bacaër N, Bravo de la Parra R, Ripoll J. Breve historia de los modelos matemáticos en dinámica de poblaciones. 2021; ISBN 9791034365883

[26] Kermack WO, McKendrick AG. A contribution to the Mathematical Theory of Epidemics. Proc. R. Soc. Lond. A. 1927; 115:700–721

[27] Sarbaz HA, Khoshnaw, Shahzad M, Ali M, Sultan F. A quantitative and qualitative analysis of the COVID-19 pandemic model. Chaos, Solitons & Fractals. 2020; 138:109932. 32523257 10.1016/j.chaos.2020.109932 PMC7247488

[28] Baba IA, Baba BA, Esmailli P. A Mathematical Model to Study the Effectiveness of Some of the Strategies Adopted in Curtailing the Spread of COVID-19. Computational and Mathematical Methods in Medicine, 2020; 2020. 10.1155/2020/5248569 PMC7556273 33082839

[29] Noutchie SC. Modeling the Spread of Covid-19 with social distancing”, Journal of Analysis & Applications. 2021; 19(1):35–46.

[30] Liu Z, Magal P, Seydi O, Webb G. Understanding Unreported Cases in the COVID-19 Epidemic Outbreak in Wuhan, China, and the Importance of Major Public Health Interventions. Biology. 2020; 9(3):50. doi: 10.3390/biology9030050 32182724 PMC7150940

[31] Pai C, Bhaskar A, Rawoot V. Investigating the dynamics of COVID-19 pandemic in India under lockdown”, Chaos, Solitons & Fractals. 2020; 138:109988. 32536763 10.1016/j.chaos.2020.109988 PMC7284270

[32] López L, Rodó X. A modified SEIR model to predict the COVID-19 outbreak in Spain and Italy: Simulating control scenarios and multi-scale epidemics. Results in Physics. 2021; 21:103746. doi: 10.1016/j.rinp.2020.103746 33391984 PMC7759445

[33] Goldsztejn U, Schwartzman D, Nehorai A. Public policy and economic dynamics of COVID-19 spread: A mathematical modelling study. PloS ONE. 2020; 15(12):e0244174. 33351835 10.1371/journal.pone.0244174 PMC7755180

[34] Lima LL, Atman APF. Impact of mobility restriction in COVID-19 superspreading events using agent-based model. PloS ONE. 2021; 16(3):e0248708. doi: 10.1371/journal.pone.0248708 33735290 PMC7971565

[35] Madubueze CE, Dachollom S, Onwubuya IO. Controlling the Spread of COVID-19: Optimal Control Analysis. Computational and Mathematical Methods in Medicine. 2020; 2020:6862516. doi: 10.1155/2020/6862516 32963585 PMC7499329

[36] Mutanga SS, Ngungu M, Tshililo FP, Kaggwa M. Systems dynamics approach for modelling South Africa’s response to COVID-19: A ‘What-If’ scenario. Journal of Public Health Research. 2021; 10(1):1897. doi: 10.4081/jphr.2021.1897 33849258 PMC7883018

[37] Ghaffarzadegan N. Simulation-based what-if analysis for controlling the spread of Covid-19 in universities”. Plos ONE. 2021; 16(2):e0246323. 33524045 10.1371/journal.pone.0246323 PMC7850497

[38] Mishra BK, Keshri AK, Rao YS, Mishra B, Mahato B, Ayesha S, et al. COVID-19 created chaos across the globe: Three novel quarantine epidemic models. Chaos, Solitons & Fractals. 2020; 138:109928. doi: 10.1016/j.chaos.2020.109928 32501378 PMC7247522

[39] Radulescu A, Williams C, Cavanagh K. Management strategies in a SEIR-type model of COVID 19 community spread. Sci Rep. 2020; 10(1):1–16. 33277553 10.1038/s41598-020-77628-4 PMC7719171

[40] Keeling MJ, Hill EM, Gorsich EE, Penman B, Guyver-Fletcher G, Holmes A, et al. Predictions of COVID-19 dynamics in the UK: Short-term forecasting and analysis of potential exit strategies. PloS Comput Biol. 2021; 17(1):e1008619. doi: 10.1371/journal.pcbi.1008619 33481773 PMC7857604

[41] Hethcote H, Zhien M, Shengbin L. Effects of quarantine in six endemic models for infectious diseases. Mathematical Biosciences. 2002; 180(1–2):141–160. doi: 10.1016/s0025-5564(02)00111-6 12387921

[42] Mishra J. A study on the spread of COVID 19 outbreak by using mathematical modelling. Results in Physics. 2020; 19:103605. 33520617 10.1016/j.rinp.2020.103605 PMC7832002

[43] Barril C, Calsina A, Cuadrado S, Ripoll J. Reproduction number for an age of infection structured model. Math. Model Nat. Phenom. 2021; 16:42.

[44] Watson GL, Xiong D, Zhang L, Zoller JA, Shamshoian J, Sundin P, et al. Pandemic Velocity: Forecasting COVID-19 in the US with a machine learning & Bayesian time series compartmental model. PloS Comput Biol. 2021; 17(3):e1008837. 33780443 10.1371/journal.pcbi.1008837 PMC8031749

[45] Baba BA, Bilgehan B. Optimal Control of a fractional order model for the COVID-19. Chaos, Solitons & Fractals. 2021; 144:110678. 33551581 10.1016/j.chaos.2021.110678 PMC7846236

[46] Gao W, Veeresha P, Baskonus HM, Prakasha DG, Kumar P. A new study of unreported cases of 2019-nCOV epidemic outbreaks. Chaos, Solitons and Fractals 2020; 138:109929. doi: 10.1016/j.chaos.2020.109929 33519103 PMC7834535

[47] Hale T, Angrist N, Goldszmith R, Kira B, Petherick A, Phillips T, et al. A global panel database of pandemic policies (Oxford COVID-19 Government Response Tracker). Nature Human Behaviour. 2021; 5(4):529–538. doi: 10.1038/s41562-021-01079-8 33686204

[48] Estrategia de detección precoz, vigilancia y control de COVID-19 de la Comunidad de Madrid https://www.comunidad.madrid/servicios/salud/coronavirus#situacion-epidemiologica-actual

[49] Estudio ENE-COVID: Informe Final. Estudio Nacional de Sero-Epidemiología de la infección por SARS-CoV-2 en España, Comunidad de Madrid, 17 de julio de 2020

[50] Estudio ENE-COVID: Cuarta Ronda. Estudio Nacional de Sero-Epidemiología de la infección por SARS-CoV-2 en España. Comunidad de Madrid, 29 de Diciembre de 2020

[51] Pollán M, Pérez-Gómez B, Pastor-Barriuso R, Oteo J, Hernán MA, Pérez-Olmeda M, et al. Prevalence of SARS-CoV-2 in Spain (ENE-COVID): a nationwide, population-based seroepidemiological study. The Lancet. 2020; 396(10250):535–544. doi: 10.1016/S0140-6736(20)31483-5 32645347 PMC7336131

[52] Pastor-Barriuso R, Pérez-Gómez B, Hernán MA, Pérez-Olmeda M, Yotti R, Oteo-Iglesias J, et al. Infection fatality risk for SARS-CoV-2 in community dwelling population of Spain: nationwide seroepidemiological study. BMJ. 2020; 371:m4509. doi: 10.1136/bmj.m4509 33246972 PMC7690290

