# Supplementary material for: COVID-19 dynamics in Madrid (Spain): A new convolutional model to find out the missing information during the first three waves
Source: PLoS One. 2022 Dec 22;17(12):e0279080. doi: 10.1371/journal.pone.0279080 (PMC9778560; doi:10.1371/journal.pone.0279080)
Supplement: S1 Text — (PDF) [file pone.0279080.s001.pdf]

COVID-19 dynamics in Madrid (Spain): a new convolutional model to find out the missing information during the first wave.

MATHEMATICAL MODEL

I. Mathematical description of the core groups

In the presented model, there are two fundamental groups which are feed by two fundamental events, the transmission and the recovery. The first will be described by a transmission rate whereas the second will be described by a convolution integral, whose mathematical descriptions start with these two definitions.

- Infected (I). Susceptible people who became infected in some past date. Although they are labelled as infected in the very moment of the infection, they are infectious only during a finite period of time.
- Recovered or dead (R). Infected people who became recovered in some past date. In this model, it is assumed that a recovered individual is not infectious anymore. This may happen because they developed immunity or because they dead. The reason is not relevant for the model since the important characteristic is that they establish a barrier to the propagation.

As long as there can be different types of infected individuals, it is necessary to give a formal description of the compartments which constitute the total number of infected individuals.

I.1 Infected individuals (transmission event)

The set of total infected individuals is $\{I\}$. As long as there are N different types of infected individuals, each type is identified with the subindex $i \in \{0,1,2,\dots,N-1\}$, being N the number of types. Thus, all the infected individuals who constitute the type i are collected in the set $\{I_i\}$. We impose this classification to be a finite partition of $\{I\}$. Therefore,

$$\{I\} = \{I_0\} \cup \{I_1\} \cup \dots \cup \{I_i\} \cup \{I_{i+1}\} \cup \dots \cup \{I_{N-1}\}$$

$$\{I_i\} \cap \left\{I_{j\neq i}\right\}I_{j\neq i} = \emptyset$$

This definition of types as a finite partition ensures that one individual is only in one type and that the number of types is finite and equal to N. These sets can be employed, for example, to classify the infected individuals attending to ensembles with specific behaviours: itinerant or non-itinerant; asymptomatic, mild, moderate, severe,…; …

In order to correctly trigger different events for each type, it is necessary to keep track of the date when the infection took place. Thus, for the infected individuals of type i, there will be individuals who were infected in the period of time between t_a and $t_a + \delta t$. Here, δt is a variable which defines the length of the period where the new infections were counted up. Assuming that $t_{a+1} = t_a + \delta t$, we can define the set of individuals who were infected in the period $[t_a, t_{a+1})$ as $\{I_i(t_a, t_{a+1})\}$. This definition lets us make a new partition:

$$\{I_i(t_0,t)\} = \{I_i(t_0,t_1)\} \cup \{I_i(t_1,t_2)\} \cup \dots \cup \{I_i(t_a,t_{a+1})\} \cup \{I_i(t_{a+1},t_{a+2})\} \cup \dots \cup \{I_i(t-\delta t,t)\}$$

$$\{I_i(t_a, t_{a+1})\} \cap \{I_j(t_b, t_{b+1})\} = \emptyset \text{ with } b \neq a$$

It is a partition because we want to avoid situations where an individual can be infected more than one time. This avoids that an individual can be infected in two different times. Thus, we call $\delta I_i(t_a) = I_i(t_a, t_{a+1}) = I_i(t_a, t_a + \delta t)$ to the number of individuals who belongs to the type i and who were infected in the period $[t_a, t_a + \delta t)$, that is, who belong to the set $\{I_i(t_a, t_{a+1})\}$. We call $I_i(t_0, t)$ to the number of individuals who are in the set $\{I_i(t_0, t)\}$. Similarly, we call $I(t_0, t)$ to the number of individuals who are in the set $\{I\}$. Thanks to having defined the sets as partitions, the following holds

$$I_i(t_0, t) = \sum_{a=0}^{n-1} \delta I_i(t_a)$$

$$I(t_0, t) = \sum_{i=0}^{N-1} I_i(t_0, t)$$

where we have used $n = (t - t_0)/\delta t$. When the number of sets tends to infinity, that is, when $n \to \infty$ and $\delta t \to 0$, the first sum leads to the following integral

$$\delta I_i(t_a) = g_i(t_a)\delta t$$

$$I_i(t_0, t) = \int_{t_0}^{t} g_i(t)dt$$

This reasoning has allowed us to define a new function $g_i(t)$, the transmission rate in the time t (towards the type-i infected), as the number of new infected individuals of type i per unit of time δt, which, for convenience, will be measured in days; and its integral $I_i(t_0, t)$, as the number of new infected individuals of type i between the times t_0 and t. Finally, the equations which rule the behaviour of the infected individuals are:

$$I_i(t_0, t) = \int_{t_0}^{t} g_i(t)dt \quad (1)$$

$$I_i(t) = I_i(t_0) + I_i(t_0, t) \quad (2)$$

$$I(t) = \sum_{i=0}^{N-1} I_i(t) \quad (3)$$

Equation (1) explicitly shows that the function of infected individuals $I_i(t_0, t)$ depends on two variables and one subindex, t_0, t and i, which respectively are the initial time, the current time and the type of new infected individuals between both times.

Equation (2) defines the number of infected individuals $I_i(t)$ at time t as the number of infected individuals $I_i(t_0)$ at time t_0 plus the number of new infected individuals $I_i(t_0, t)$ in the period between t_0 and t. Therefore, with this nomenclature, the function with one argument collects the number of infected individuals from the onset while the function with two arguments collects the number of infected individuals between two different times.

Equation (3) defines the total number of infected individuals $I(t)$ at time t as the sum of all the infected individual $I_i(t)$ of each type i.

The most important equation is (1) because it allows us to label all the infected individuals with the date when they became infected. For example, the number of all the new infected individuals between τ and $\tau + \delta\tau$ is $I_i(\tau, \tau + \delta\tau) = \int_{\tau}^{\tau+\delta\tau} g_i(t)dt$, which, when $\delta\tau$ tends to zero, allows us to obtain the Equation (4), which records the individuals who were new infections just in the time τ.

$$\delta I_i(\tau) = g_i(\tau)\delta\tau \tag{4}$$

Keeping the initial time t_0 with a constant value, Equation (1) leads also to

$$\frac{\partial I_i(t_0, t)}{\partial t} = g_i(t) \tag{5}$$

As long as time t_0 keeps constant, Equation (2) lets also write Equation (5) as

$$\frac{dI_i(t)}{dt} = g_i(t) \tag{6}$$

This is a set of differential equations which describe the transmission events. Functions $g_i(t)$ will be fully described in section III and explicitly written in Equation (12).

I.2 Recovered individuals (recovery event)

The recovery event happens when an infected individual becomes a non-infectious one. This happens after a period of time Δt; however not all the infected individuals were infected in the same date and not all of them requires the same period of time to be recovered.

Equation (4) gives the individuals who were infected just in the past time τ as $\delta I_i(\tau)$. In the current time t, these individuals have suffered the disease during the period of time $\Delta t = t - \tau \geq 0$. As long as t increases, a greater fraction of them will be recovered. Let us call $F(\Delta t; \boldsymbol{PM_{Ri}})$ to the fraction of infected individuals who were infected at the same time and that, after having lived with the illness during the period of time Δt, are now recovered. Note that here $\boldsymbol{PM_{Ri}}$ is a set of parameters (for example, the median and the IQR) which identify that the recovery event is different for each type i. As long as the time τ is in the past, $\delta I_i(\tau)$ does not change while t increases. Hence, the number of recoveries after the period Δt is $F(\Delta t; \boldsymbol{PM_{Ri}})\delta I_i(\tau) = F(t - \tau; \boldsymbol{PM_{Ri}})g_i(\tau)d\tau$. However, at time t, there must be recovered individuals who were infected at different times τ.Taking into account all the individuals who were infected between the times t_0 and t we obtain the equations which rule the behaviour of the recovered individuals as

$$R_i(t_0, t) = \int_{t_0}^{t} F(t - \tau; \boldsymbol{PM_{Ri}})g_i(\tau)d\tau \tag{7}$$

$$R_i(t) = R_i(t_0) + R_i(t_0, t) \tag{8}$$

$$R(t) = \sum_{i=0}^{N-1} R_i(t) \tag{9}$$

The main advantages of this model come from Equation (7), which is a convolution integral between the transmission rate $g_i(\tau)$ and the fraction of expected recoveries $F(\Delta t; \boldsymbol{PM_{Ri}})$. This integral makes the model more flexible because:

1. The recovery event has uncertainty in the required number of days.
2. This uncertainty depends only on the type of infection, being different for each type i.
3. The parameters $\boldsymbol{PM_{Ri}}$ defining this uncertainty have a clear meaning (median and IQR, or any other set which the manager had available).

If the initial time t_0 is fixed, Equation (7) can be derived to obtain

$$\frac{\partial R_i(t_0,t)}{\partial t} = g_i(t)F(0;\boldsymbol{PM_{Ri}}) + \int_{t_0}^{t} g_i(\tau)f(t-\tau;\boldsymbol{PM_{Ri}})d\tau$$

Here $f(\Delta t;\boldsymbol{PM_{Ri}})$ is the probability density function defined as

$$f(\Delta t;\boldsymbol{PM_{Ri}}) = \frac{dF(\Delta t;\boldsymbol{PM_{Ri}})}{d\Delta t}$$

We assume that an event always happens if the time is long enough, and for such reason $f(t)$ will accomplish the following relation

$$\int_0^{\infty} f(\tau;\boldsymbol{PM_{Ri}})d\tau = 1$$

It is plausible to assume that the fraction of recoveries after a null period of time is zero, $F(0;\boldsymbol{PM_{Ri}}) = 0$, what allows us to simplify the differential equation to:

$$\frac{\partial R_i(t_0,t)}{\partial t} = \int_{t_0}^{t} g_i(\tau)f(t-\tau;\boldsymbol{PM_{Ri}})d\tau \tag{10}$$

This is a set of differential equations which describe the recovery events and depend on the functions $g_i(t)$ and $f(\Delta t;\boldsymbol{PM_{Ri}})$ which will be respectively described in sections III and V. What is interesting here is that the convolution integral uses exactly the same transmission function $g_i(t)$ which appears in Equation (5) to describe the transmission event. For this reason, all the relevant information regarding the recovery event as a health issue is exclusively collected in $f(\Delta t;\boldsymbol{PM_{Ri}})$. This ensures that transmission and recovery events can be defined independently: each event can be completely defined without taking into account the other because the convolution integral is in charge of mixing them correctly in the mathematical description.

As long as time t_0 keeps constant, Equation (10) lets also write Equation (11) as

$$\frac{dR_i(t)}{dt} = \int_{t_0}^{t} g_i(\tau)f(t-\tau;\boldsymbol{PM_{Ri}})d\tau \tag{11}$$

Taking into account Equation (6) and dropping the subscript for the type of infectious in Equation (11), we recover the simplified C-SIR model which appears in the main article:

$$\frac{dR(t)}{dt} = \int_0^{t} f(t-\tau)\frac{dI(\tau)}{d\tau}d\tau$$

Here, the distribution function $f(t)$ fixes the probability that an event has of happening just at time t as $f(t)dt$ and, since the event described is the recovery or death, $f(t-\tau)d(t-\tau) = f(t-\tau)d(t)$ is the recovery or death of the infected individuals who were infected at time τ

and have lived with the disease during the time $t-\tau$. That is, if there were $dI(\tau)$ new infected individuals in the interval of time $[\tau, \tau+d\tau)$ then $dI(\tau)f(t-\tau)dt$ would be the number of them who became recovered or dead during the interval of time $[t,\ t+dt)$. However, any particular time τ in the interval $[0,\ t)$ can lead to recovered or dead individuals during the interval of time $[t,\ t+dt)$. For this reason, the total number of recovered or dead individuals $dR(t)$ during the interval of time $[t,\ t+dt)$ is $dR(t)=\int_0^t dI(\tau)f(t-\tau)dt$, which after some minor algebra leads to the previous Equation. Although there are a multitude of candidates for the distribution function $f(t)$, we choose a Weibull distribution because it is versatile enough and has some mathematical advantages. Weibull distribution has two parameters which can be selected to set the median and the IQR to the desired values (see section V).

II. Mathematical description of non-core groups related to the transmission event

The two core groups described in section I allow us to define other groups; the most important ones for describing the transmission events are:

- Carriers ($C=\alpha\, Inf$). Infectious people without restrictions in their mobility.
  - Infectious individuals ($Inf=I-R$). Infected people who is not recovered yet.
- Susceptible individuals ($S=P-I$). Healthy people without restrictions in their mobility.
  - Accessible population ($P=\chi P_T$). People who is accessible to the infection event. Here, P_T is the total number of individuals in the studied population and χ is the accessible fraction.

Next sections define them attending to the fundamental groups.

II.1 Carriers

Every infected individual who is not recovered can propagate the disease. However, many of them can have restrictions in their mobility. Hence, we define the carriers as those infectious individuals whose mobility is not restricted. Mathematically, the number of carriers who belong to the type i is:

$$C_i(t)=\alpha_i(t)[I_i(t)-R_i(t)]$$

In this expression, the factor $\alpha_i(t)\in[0,1]$ has into account that there are infectious individuals who cannot propagate the disease. This way, when α_i is near 0, almost all the infectious individuals are isolated and cannot meet other individuals (they are in adequate hospital areas, confined to their homes, etc.) whereas when α_i is near 1, almost all of them are free to propagate the virus. Therefore, α_i is defined as the fraction of infectious people of type i who are free to move through the population or, equivalently, to the fraction of time which an infectious individual shares with accessible people. Thus, a first estimation of α_i comes from taking into account that infectious individuals can only freely move during the first part of the infection period, for example, before being admitted to hospital.

II.2 Susceptible individuals

The individuals who are susceptible to being infected are all the accessible individuals who have not been infected yet. Thus, at time t, the susceptible individuals are the accessible individuals minus the infected individuals:

$$S(t) = \chi(t)P_T - I(t)$$

Obviously, the model requires the condition $P(t) = \chi(t)P_T > I(t)$, where $P(t)$ is the accessible population. The accessible population is composed of both, susceptible and infected. Susceptible individuals are healthy people who can be infected (here, it is not relevant if they are recovered or not). This definition is important because, for example, the vaccinated cannot belong to the accessible population, what indicates that $P(t)$ must be less than the total population P_T. Another case where $P(t) < P_T$ appears is when the total population P_T contains population pockets (for example, isolated towns) that are out of the path of transmission but that eventually can be incorporated to such path. This implies that, in general, the accessible population is a function of time. Thus, $\chi(t) = P(t)/P_T \in [0,1]$ is the fraction of accessible population at time t.

III. Mathematical description of the transmission event

The carriers and the susceptible individuals are the agents involved in the propagation of the disease. The susceptible population can be infected due to the movements of the internal carriers or due to the movements of foreign individuals or healthy travellers who returns as infected. Next sections describes both situations.

III.1 Transmission for internal contacts

The internal contact happens when a carrier inside the population contacts with a susceptible who is also inside the population. We assume that the number of new infections of type j provoked by a type-i infectious is proportional to the number of carriers $C_i(t)$ of such type and to the frequency $\omega_{ij}(t)$ (its units are 1/day) Therefore, the function $g_j(t)$ which appears in equations (6) and (11) is given by

$$g_j(t) = \sum_{i=0}^{N-1} C_i(t)\omega_{ij}(t)$$

This equation has into account that all the types $i = 1, 2, \dots N$ can produce new type-j infected individuals. To complete the model, it is necessary to include in the frequency $\omega_{ij}(t)$ the following influences:

a. The average frequency $\omega(t)$, which is the average number of people that an average person contact per day (its units are person/person/day).
b. The factor $\gamma_i(t)$, which measures the average success in the contact that an infected of type i has.
c. The factor $\phi_j(t)$, which is the fraction of infected individuals who are going to be of type j . Note that $\sum_{j=0}^{N-1} \phi_j(t) = 1$ holds.
d. The fraction $S(t)/P(t)$., which takes into account the probability of finding a susceptible inside the population.

Therefore, these conditions let write the frequency as

$$\omega_{ij}(t) = \omega(t)\frac{S(t)}{P(t)}\gamma_i(t)\phi_j(t)$$

III.2 Transmission for external contacts

The external contact happens in either of these two cases:

1. When a healthy person inside the population travels abroad and meets individuals in other external populations.
2. When an infected person outside the population travels inside the population and meets individuals in the population.

In the first case the probability of being infected abroad as a type j is proportional to

$$\beta_A(t)S(t)\omega(t)\phi_j(t)\sum_{i=0}^{N-1}\frac{\gamma_i(t)C_i(t)}{P(t)} = \beta_A(t)\sum_{i=0}^{N-1}C_i(t)\omega_{ij}(t)$$

The explanation of this expression is as follows. The number of healthy travellers who daily cross the border is $\beta_A(t)S(t)$. One of these travellers has the probability $\phi_j(t)$ of belonging to type j. This traveller finds $\omega(t)$ persons per day and, among this persons, the fraction $\frac{C_i(t)}{P(t)}$ are carriers of type i with a success in the transmission γ_i; as a consequence, the frequency of transmission is proportional to $\sum_{i=0}^{N-1}\frac{\gamma_i(t)C_i(t)}{P(t)}$.

In the second case, the probability of being a traveller of type i is

$$\frac{S(t)}{P(t)}\omega(t)\phi_j(t)\sum_{i=0}^{N-1}\beta_B(t)\gamma_i(t)C_i(t) = \beta_B(t)\sum_{i=0}^{N-1}C_i(t)\omega_{ij}(t)$$

The explanation of this expression is as follows. The number of travellers per day (infected as type i) who cross the border is $\beta_B(t)C_i(t)$, with a success in the transmission γ_i. Thus, the fraction of travellers who daily comes with capacity to transmit the disease is $\sum_{i=0}^{N-1}\beta_B(t)\gamma_i(t)C_i(t)$. One of this infected travellers can meet $\omega(t)$ individuals per day, but only the fraction $\frac{S(t)}{P(t)}$ is susceptible of being infected as type j with probability $\phi_j(t)$.

It is necessary to observe that in both cases we have used $\frac{\gamma_i(t)C_i(t)}{P(t)}$ as the fraction of carriers of type i, what is equivalent to say that the travellers abroad have the same distribution of carriers as the studied population and that such carriers have the same success to transmit the disease inside or abroad. The unique difference between both cases is due to the fractions $\beta_A(t)$ and $\beta_B(t)$, which respectively represent the fraction of population who comes from outside or who goes outside. Therefore, we can define a new function $\beta(t) = \beta_A(t) + \beta_B(t)$ as the people who crosses the border in either direction. This way, a population where all its individuals daily cross the border has double probability of being infected. Obviously, this extreme case is not real because in such situation the studied population would have wrongly selected; it should be expanded to include the population where these travellers mix. For these reason, $\beta(t)$ is expected to be a small positive number.

In addition, governments can impose restrictions to the mobility of some population pockets. This will lead to a negative value of $\beta(t)$, with its lower value being -1. This way $\beta(t)$ can be seen as a stringency index which measures how strong social limitations are.

III.3 Transmission for both internal and external contacts

The total number of new infections is the addition of both, internal and external, which the previous sections has described as

$$g_j(t) = (1 + \beta(t)) \sum_{i=0}^{N-1} C_i(t)\omega_{ij}(t)$$

This way, the external contact may be understood as an increment of the internal contact $\omega(t)$ in such a way that it becomes $(1 + \beta(t))\omega(t)$ instead of $\omega(t)$. Therefore, the final equation to calculate the function $g_i(t)$ in terms of the core groups $I_i(t)$ and $R_i(t)$ is

$$g_i(t) = \phi_i(t)[1 + \beta(t)]\omega(t) \left[1 - \sum_{i=0}^{N-1} \frac{I_i(t)}{\chi(t)P_T}\right] \sum_{j=0}^{N-1} \alpha_j(t)\left[I_j(t) - R_j(t)\right] \gamma_j(t) \qquad (12)$$

IV. Mathematical description of non-core groups related to other health events

Equations (6), (11) and (12) are the core equations which allow us to solve the core events and groups. However, although the essential events are the transmission and the recovery previously described, the health management requires knowing the evolution of other groups and events such as vaccination, deaths, admissions and discharges from hospitals and ICUs, etc. This section describes the ones used in the presented article: covid-case confirmation, admission in hospital, admission in ICU and death.

IV.1 Covid-case confirmations

Covid-case confirmation is a stochastic event which can be described by a convolution integral such as

$$\frac{dConf_i(t)}{dt} = \psi_{Ci}(t) \int_{t_0}^{t} g_i(\tau) f(t - \tau; \boldsymbol{PM_{Ci}}) d\tau \qquad (13)$$

where $\psi_{Ci}(t)$ is the fraction of new infected which will be confirmed as positive ones. Each infected individual with symptoms will be tested to discover whether it is a covid-case or not, and for this reason it is expected to have a fraction $\psi_{Ci}(t)$ equal to 1. In the case of asymptomatic individuals, a small fraction of them will be discovered by the government's systematic screenings, and $\psi_{Ci}(t)$ will be less than 1 since not all the asymptomatic infections will be detected.

IV.2 Admission in hospital

Admission in hospital is a stochastic event which can be described by a convolution integral. Each moderate or severe infected individual will, for sure, be admitted in a hospital whereas asymptomatic and mild infections will not, for sure, be admitted. The equations to obtain the daily new admissions are

$$\frac{dHosp_i(t)}{dt} = \psi_{Hi}(t) \int_{t_0}^{t} g_i(\tau) f(t-\tau; \boldsymbol{PM_{Hi}}) d\tau \tag{14}$$

IV.3 Admission in ICU

Admission in ICU is a stochastic event which can be described by a convolution integral. Each severe infected individual will, for sure, be admitted in a hospital whereas asymptomatic mild and moderate infections will not, for sure, be admitted. The equations to obtain the daily new admissions are

$$\frac{dICU_i(t)}{dt} = \psi_{ICUi}(t) \int_{t_0}^{t} g_i(\tau) f(t-\tau; \boldsymbol{PM_{ICUi}}) d\tau \tag{15}$$

IV.4 Deaths

Death is an instant event that a fraction of recovered individuals suffer. It is plausible that each type has a different fatality. Severe infections will present a larger fraction than moderate ones. Let ψ_{Di} be the fraction of recoveries of type i that are deaths, thus the daily number of deaths becomes

$$\frac{dDef_i(t)}{dt} = \psi_{Di}(t) \frac{dR_i(t)}{dt} \tag{16}$$

V. Mathematical description of the probability density function

The complete convolutional model proposed in this article uses Equations (6), (11) and (12) to (16) to calculate the temporal evolution of the defined groups. In the model, Equations (11) and (13) to (15) have convolutions integrals which require to know a probability distribution function $f(t-\tau; \boldsymbol{PM_x})$. As can be seen, the function is the same in all the equations and what changes is the set of parameters used $\boldsymbol{PM_X}$ to feed it. This set is different depending on the event X which is being described.

This section develops the density function used in this article, which is a Weibull distribution. We have found that the Weibull distribution is accurate enough; however, since the proposed convolutional model is defined for any general distribution function, reader may use any other custom distribution. In the particular case presented in this study, the probability density function is

$$f(\Delta t, \boldsymbol{PM}) = \frac{1}{m\mu} \left(\frac{\Delta t}{\mu}\right)^{m-1} e^{-\left(\frac{\Delta t}{\mu}\right)^m}$$

$$\boldsymbol{PM} = [\mu, m]$$

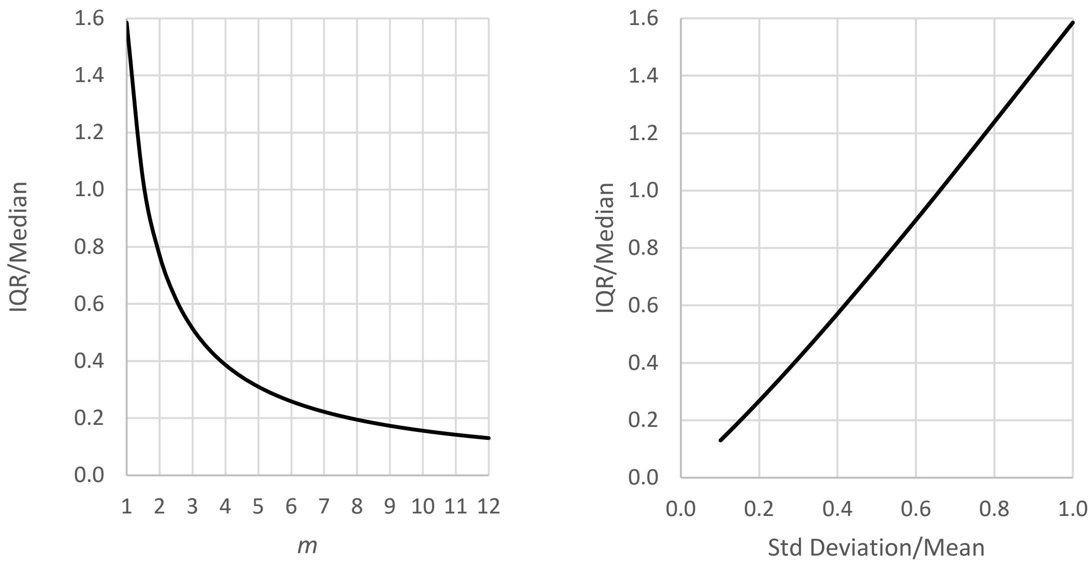

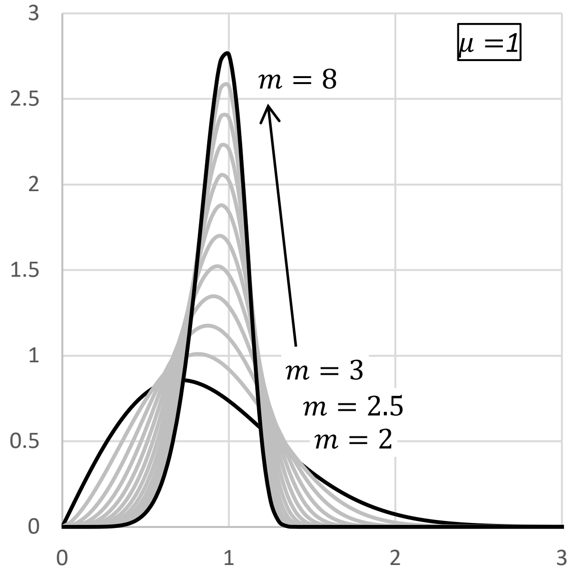

Figure 1. Relation between the parameters which determine the shape of the probability distribution and the distribution function itself. The bottom-left graph shows how the parameter m affects to the shape of the distribution. The top-left graph shows how the parameter m determines the relation between the IQR and the median. The top-right graph shows how the standard deviation and the mean determine the IQR/Median ratio.

Note that the parameters $[\mu, m]$ can be obtained from the median and the IQR using the following equations

$$m = \frac{1}{2}\left[m + \frac{\ln 2}{\ln\left(\frac{IQR}{median} + \left(\frac{\ln 4/3}{\ln 2}\right)^{\frac{1}{m}}\right)}\right]$$

$$\mu = \frac{median}{(\ln 2)^{1/m}}$$

In particular, first equation can be solved in the range $IQR/Median \epsilon [0.1, 1.5]$ using an iterative procedure with the seed m =12. Figure 1 shows the relation between the IQR/Median ratio and the parameter m and the Standard Deviation/Mean ratio.

VI. Describing social and health changes

The events which occur in the society at some discrete times t_E (such as lockdowns, restoration of mobility, use of facial masks, etc.) can be taken into account by changing the values of the constants at such times: $\beta(t_E)$, $\omega(t_E)$, $\chi(t_E)$, $\phi_i(t_E)$, $\alpha_i(t_E)$, $\gamma_i(t_E)$, $\psi_{Di}(t_E)$ and $\boldsymbol{PM_{Ri}}(t_E)$ = [$\mu_{Ri}(t_E), m_{Ri}(t_E)$]. Choosing adequately the value of these constants during special periods (for example, during a lockdown) allows us to include complex scenarios in the model and compare the result of applying different containment and propagation alternatives.

- $\chi(t_E)$ is the fraction of population which is under study. This value can be increased, for example, to have into account that some regions which were isolated are not isolated anymore. It could be decreased to take into account that a part of the population has been vaccinated.
- $\alpha_i(t_E)$ is the proportion of carriers of type i who can propagate the disease. Its value is between 0 and 1. A value near 0 describes a scenario where the mobility of this type of infected individuals has been controlled, for example, because they are in a hospital with enough measures of protection. A value near 1 describes a scenario where all the carriers has maximum freedom to move, for example, because they are asymptomatic individuals who continue with their normal life. In general, it should be expected a lower mobility for severe than for mild cases.
- $\omega(t_E)$ is the number of persons that an average person whose mobility has not been restricted found on average per day. This parameter has into account how the persons relate each other. A scenario with a general lockdown could reduce this value; however, it is better to use the next parameter for this use.
- $\beta(t_E)$ is the fraction of the population who is traveling across the border. It allows to describe regions with airports, etc. When the airports are closed or when the borders are closed, its value decreases. A value of $\beta(t_E)$ equals to -1 means that the region has been completely paralyzed. Since this parameter increases or reduces the effective value of ω it can be used instead of it. This has been the case in the presented study.
- $\gamma_i(t_E)$ is the fraction of healthy persons who an infected of type i can infect when is physically near them. It has into account the efficiency of the transmission channel: a very effective channel will have a value near 1, whereas a very inefficient channel will have a value near 0. Its effect is identical to α_i, but its cause is different: for example, using masks and frequently washing hands reduce the efficiency of the channel although the mobility of the infectious keeps the same.
- $\phi_i(t_E)$ measures the population's propensity to develop a mild or a severe disease. It must verify $\sum_{i=0}^{N-1} \phi_i(t_E) = 1$. If the probability of having severe infections increases, the probability of having other types decreases.
- $\psi_{Di}(t_E)$ is the fraction of recovered individuals who dead. It decreases, for example, if medical procedures improves.
- $m_{Ri}(t_E)$ defines the shape of the probability distribution. A low value means that there is a huge dispersion or uncertainty. The other way round, a large certainty requires a larger value of this parameter.
- $\mu_{Ri}(t_E)$ defines the temporal scale for the convolutional events. The larger the value, the larger the delay between the trigger signal and the occurrence of the event.

The temporal influence of all those effects which are related to mobility can be lumped together into one single parameter: in this model, without any loss of generality, the stringency index $\beta(t_E)$ has been selected for this purpose.
